# Supplementary material for: Sexual orientation identity and tobacco and hazardous alcohol use: findings from a cross-sectional English population survey
Source: BMJ Open. 2017 Oct 25;7(10):e015058. doi: 10.1136/bmjopen-2016-015058 (PMC5665254; doi:10.1136/bmjopen-2016-015058)
Supplement: Author's manuscript [file bmjopen-2016-015058.draft_revisions.pdf]

BMJ Open is committed to open peer review. As part of this commitment we make the peer review history of every article we publish publicly available.

When an article is published we post the peer reviewers' comments and the authors' responses online. We also post the versions of the paper that were used during peer review. These are the versions that the peer review comments apply to.

The versions of the paper that follow are the versions that were submitted during the peer review process. They are not the versions of record or the final published versions. They should not be cited or distributed as the published version of this manuscript.

BMJ Open is an open access journal and the full, final, typeset and author-corrected version of record of the manuscript is available on our site with no access controls, subscription charges or pay-per-view fees (http://bmjopen.bmj.com).

If you have any questions on BMJ Open's open peer review process please email editorial.bmjopen@bmj.com

BMJ Open

Sexual orientation identity and tobacco and hazardous alcohol use: findings from a cross-sectional English population survey

Journal:	*BMJ Open*
Manuscript ID	bmjopen-2016-015058
Article Type:	Research
Date Submitted by the Author:	08-Nov-2016
Complete List of Authors:	Shahab, Lion; UCL, Dept of Epidemiology and Public Health<br>Brown, Jamie; University College London, Psychology & Language Sciences<br>Hagger-Johnson, Gareth; UCL, Farr Institute<br>Michie, Susan; University College London, Centre for Outcomes Research and Effectivenes<br>Semlyen, Joanna; University of East Anglia Faculty of Medicine and Health Sciences<br>West, Robert; University College London, Epidemiology and Public Health<br>Meads, Catherine; RAND Europe
**Primary Subject Heading**:	Smoking and tobacco
Secondary Subject Heading:	Addiction, Epidemiology, Public health
Keywords:	EPIDEMIOLOGY, Health policy < HEALTH SERVICES ADMINISTRATION & MANAGEMENT, Substance misuse < PSYCHIATRY, PUBLIC HEALTH

SCHOLARONE™
Manuscripts

For peer review only

Sexual orientation identity and tobacco and hazardous alcohol use: findings from a cross-sectional English population survey.

Lion Shahab[1*], Jamie Brown[1,2], Gareth Hagger-Johnson[3], Susan Michie[2], Joanna Semlyen[4], Robert West[1], Catherine Meads[5]

[1] Department of Epidemiology and Public Health, University College London, London, UK

[2] Department of Clinical, Educational and Health Psychology, University College London, London, UK

[3] Administrative Data Research Centre for England (ADRC-E), Farr Institute, University College London, London, UK

[4] Norwich Medical School, University of East Anglia, Norwich, UK

[5] RAND Europe, Cambridge, UK

*Correspondence to: Dr Lion Shahab, Department of Epidemiology & Public Health, University College London, 1–19 Torrington Place, London, WC1E 6BT, UK; Phone: +44 207679 1805; Fax: +44 207813 2848; Email: lion.shahab@ucl.ac.uk

Abstract: 273/300

Manuscript: 3512/4000 (excluding abstract, figures, tables, legends and references)

Figures and tables: 5/5

References: 47

For peer review only - http://bmjopen.bmj.com/site/about/guidelines.xhtml

Abstract

Objectives: To assess the association between tobacco and hazardous alcohol use and sexual orientation and whether such an association could be explained by other socio-demographic characteristics.

Design: Cross-sectional household survey conducted in 2014-2016.

Setting: England, UK.

Participants: Representative English population sample (pooled N=43,866).

Main Outcomes: Sexual orientation identity (lesbian/gay/bisexual (LGB), heterosexual, prefer-not-to-say); current tobacco and hazardous alcohol use (defined as Alcohol Use Disorders Identification Test Score ≥ 8). All outcomes were self-reported.

Results: Due to interactions between sexual orientation and gender for substance use, analyses were stratified by gender. Tobacco use prevalence was significantly higher among lesbian/gay (women: 24.9%, 95% confidence interval (CI) 19.2%-32.6%; men: 25.9%, 95%CI 21.3%-31.0%) and bisexual participants (women: 32.4%, 95%CI 25.9%-39.6%; men: 30.7%, 95%CI 23.7%-30.7%) and significantly lower for prefer-not-to-say participants in women (15.5%, 95%CI 13.5%-17.8%) but not men (22.7%, 95%CI 20.3%-25.3%) compared with heterosexual participants (women: 17.5%, 95%CI 17.0%-18.0%; men: 20.4%, 95%CI 19.9%-21.0%; all p<0.001). Similarly, hazardous alcohol use was significantly more prevalent for lesbian/gay (women: 19.0%, 95%CI 14.0%-25.3%; men: 30.0%, 25.2%-35.3%) and bisexual participants (women: 24.4%, 95%CI 18.7%-31.3%; men: 24.3%, 95%CI 17.9%-32.1%) and lower for prefer-not-to-say participants (women: 4.1%, 95%CI 3.0%-5.4%; men: 13.7%; 95%CI 11.8-16.0%) compared with heterosexuals (women: 8.3%, 95%CI 7.9%-8.7%; men: 18.4%, 95%CI 17.9%-18.9%; all p<0.001). However, after adjusting for socio-demographic confounders, tobacco use was similar in all groups, whereas for hazardous alcohol use all differences remained except for bisexual men.

Conclusions: In England, higher rates of tobacco use found among sexual minority men and women appear to be attributable to other socio-demographic factors. Higher rates of hazardous alcohol use among lesbian and bisexual women and gay men persist after adjustment for other sociodemographic factors.

Key words: Tobacco use, hazardous alcohol use, sexual orientation identity, LGB, epidemiology

For peer review only - http://bmjopen.bmj.com/site/about/guidelines.xhtml

Strengths and limitations of this study

- Research suggests that substance use is more prevalent among some sexual minorities. However, most research in this area involves small convenience samples and data from representative samples is scarce.
- This study is one of only very few studies to investigate this issue in a large representative population survey, controlling for a wide range of confounders, to assess the association between tobacco and hazardous alcohol use and sexual orientation identity with the aim to inform health policy, allocate resources and, if necessary, develop targeted interventions.
- As is the case for all cross-sectional analysis we cannot infer causality between substance abuse and sexual orientation, and the measure of sexual orientation may not have captured all dimensions of this construct.

For peer review only

For peer review only - http://bmjopen.bmj.com/site/about/guidelines.xhtml

Introduction

Knowledge about the health behaviours of sexual minority (lesbian, gay, and bisexual, (LGB)) groups is necessary for monitoring health inequalities, developing public health policies, allocating resources and targeting high-risk groups for interventions [1 2].

There is a validated measure of sexual orientation developed by the Office of National Statistics [3] but despite recent equality and diversity legislation and the inclusion of sexual orientation in the National Health Service (NHS) Equality Delivery System, sexual orientation identity is not regularly monitored and/or reported by NHS organisations or collected in epidemiological research. This may in part be due to the complex nature of sexual orientation, which can be defined and assessed along dimensions of behaviour, identity or attraction, or a combination of these [4]. The different dimensions of identity, behaviour and attraction do not map easily onto each, and even within these dimensions, not all possible manifestations have been explored [5]. Other methodological issues include the fact that, historically, sexual minorities were relatively more hidden due to stigmatisation, resulting in, small, non-representative samples being studied [6].

As a result, the evidence base on health inequalities experienced by sexual minorities is sparse and has only recently begun to be explored. In the UK, LGB sexual identity and behaviour has been assessed in the National Attitudes of Sexual Attitudes and Lifestyles (NATSAL; 1990–1991, 1999–2001, 2010–2012), National Statistics Opinions Survey (in 2008–2009), Office for National Statistics (ONS) Integrated Household Survey (from 2009), Longitudinal Study of Young People in England (from 2009), British Cohort Study (2012), Health Survey for England (from 2010), Scottish Health Survey (from 2008), English Longitudinal Study of Ageing (2012/13) and Understanding Society (from 2011/12). In addition, several major cohort studies in the USA have included questions on sexual orientation identity in recent years [7 8]. Yet, due to the methodological difficulties discussed above, most evidence on health behaviours so far has come from small convenience samples, suggesting that sexual minorities are more likely to smoke tobacco

For peer review only

or engage in harmful alcohol use [8]. Representative population data across the entire adult age range, especially from non-US samples, are scarce [5 9 10].

Several possible mechanisms have been proposed that connect minority sexual orientation identity to health behaviours such as smoking and alcohol use. The concept of ‘minority stress’ is often invoked to explain how heterosexism and homophobia are internalised [11], perhaps leading people to self-medicate for psychological distress with cigarettes or other substances such as alcohol. A recent review found support for this theory, particularly in explaining associations with victimisation and substance use [12]. Another factor could be lower levels of self-esteem, wellbeing and greater propensity to mental health problems in this population brought on by biases towards sexual minorities [5 6 13] which in themselves have been linked to tobacco and alcohol abuse [14 15]. Alternatively, LGB young people, in particular, may be more likely to access adult venues where alcohol and cigarettes are easily available. Due to a lack of social support at school, from peers and family, LGB young people may seek this support and socialise in recreational spaces where peer norms encourage engagement in risky health behaviours [16], or these behaviours may be adopted in order to appear older to gain access to a venue or to fit in [17]. Substance use may be sexually arousing for some individuals and subcultures, particularly when seen to signify masculinity [18]. Other commentators have noted the role of industry in targeting this community [19]. It is also possible that apparent differences in prevalence may be an artefact of other socio-demographic differences associated with sexual minorities or the use non-representative data.

There is clear need for population level studies to investigate the extent of the problem further. This will help reduce health inequalities for the LGB community by better describing tobacco and alcohol use behaviour and by identifying drivers for change. This study aimed to assess the prevalence and association of tobacco and hazardous alcohol use with sexual orientation identity. Specifically, the study addressed the following questions:

For peer review only - http://bmjopen.bmj.com/site/about/guidelines.xhtml

1) What is the prevalence of tobacco and hazardous alcohol use in a large LGB population sample? Does it differ by gender or from the heterosexual population?

2) Are differences in tobacco and hazardous alcohol use, if any, between the LGB and heterosexual population sample attenuated when controlling for other sociodemographic covariates?

For peer review only

For peer review only - http://bmjopen.bmj.com/site/about/guidelines.xhtml

Methods

Study design and participants

Data were collected using cross-sectional household surveys of representative samples of the population of adults in England. The surveys are part of the ongoing series of Smoking and Alcohol Toolkit Studies, designed to provide tracking information about smoking, alcohol consumption and related behaviours in England. Each month a new sample of approximately 1,700 adults aged ≥16 years complete a face-to-face computer-assisted survey with a trained interviewer (see [20 21] for full details). Current data were collected from March 2014 until May 2016 where complete data on tobacco, alcohol use, sexual orientation and other baseline socio-demographic variables were included. A total of 45,423 adults were surveyed in this time period, of whom 43,886 had complete data. The study received ethical approval from the University College London Research Ethics Committee and was carried out in accordance with the ethical principles on human research, as set out in the Declaration of Helsinki.

Measures

Sexual Identity

Sexual orientation was assessed by asking respondents to self-identify as (a) Bisexual; (b) Gay man/homosexual; (c) Gay woman/lesbian; (d) Heterosexual/straight; (e) Prefer not to say. This measure deviates slightly from the recommended ONS item (which also provides an 'Other' option) [3] but produces similar prevalence rates compared with the latest ONS Integrated Household Survey [22] (see Table 1).

Table 1: Prevalence of self-perceived sexual orientation identity in the Smoking and Alcohol Toolkit Study 2014-2016 and ONS Integrated Household Survey, 2014

	Toolkit Study	ONS
	% (95% Confidence Interval)	
Heterosexual/Straight	93.3 (93.0-93.5)	92.8
Bisexual	0.7 (0.6-0.8)	0.5
Lesbian/Gay	1.1 (1.0-1.2)	1.1
PNTS	4.9 (4.7-5.1)	5.3
Other	-	0.3

PNTS – Prefer-not-to-say

For peer review only - http://bmjopen.bmj.com/site/about/guidelines.xhtml

Tobacco use

To assess tobacco use, participants were asked if they (a) smoked cigarettes (including hand-rolled) every day; (b) smoked cigarettes (including hand-rolled) but not every day; (c) did not smoke cigarettes at all but did smoke tobacco of some kind (e.g. pipe or cigar); (d) had stopped smoking completely in the last year; (e) had stopped smoking completely more than a year ago; or (f) had never been a smoker (i.e. smoked for a year or more). Current smokers were classified as answering 'yes' to (a) to (c), and ex-smokers, classified as answering 'yes' to (d) or (e). Those answering 'yes' to (f) were classified as never-smokers and together with ex-smokers also classified as non-smokers. Additionally, current smokers were asked questions to determine nicotine dependence (measured by heaviness of smoking index, (HSI) [23], and strength of urges to smoke, (SUTS) [24]) as well as motivation to quit (measured by the motivation to stop scale, (MTSS) [25]). Smokers also provided an estimate of money spent per week on tobacco products, whether they had made at least one serious quit attempt in the last year and whether they had been asked about their smoking, received advice and/or support to stop from their GP.

Alcohol use

Alcohol use was assessed with the well-established and reliable Alcohol Use Disorders Identification Test (AUDIT) questionnaire, with a cut-off ≥ 8 to indicate hazardous drinking [26]. Participants who scored as hazardous drinkers were further asked about their urges to drink (adapted from the SUTS) and their motivation to cut down alcohol consumption (adapted from the MTSS). They were also asked how much money they spent per week on alcohol for their own consumption, whether they had made a serious attempt to cut down in the last year and whether they had been asked about their drinking, received advice and/or support to cut down from their GP.

Covariates

Standard socio-demographic characteristics assessed included age (in years), sex (male/female), ethnicity (white/non-white), marital status (married, civil partnership or living with partner; yes/no), socio-

For peer review only - http://bmjopen.bmj.com/site/about/guidelines.xhtml

economic status; (ABC1/C2DE), disability; (yes/no), education (post-16 qualification; yes/no), England region; (North/Central/South) and internet use; (>daily/≤daily/never).

Analysis

Data were analysed with IBM SPSS Statistics Version 22.0. All prevalence and descriptive data were weighted using the rim (marginal) weighting technique to match an English population profile on the dimensions of age, social grade, region, tenure, ethnicity and working status within sex derived from the English 2011 census, ONS 2013 mid-year estimates and a random probability survey conducted in 2014 for the National Readership Survey [27]. Further details on the weighting procedure are reported elsewhere [28]. Simple associations between study groups and continuous socio-demographic variables, tobacco use and alcohol use characteristics were assessed with one-way ANOVAs, and categorical variables were assessed with chi-square analysis or logistic regression. We included interaction terms for sexual orientation by gender in the logistic regressions that assessed the main binary outcomes (tobacco and hazardous alcohol use) to determine if there were systematic differences. Family-wise error rate was corrected using the false discovery rate [29] and multiple comparisons were controlled for using the Sidak correction in post-hoc analysis.

For peer review only

Results

Table 2 provides a breakdown of socio-demographic characteristics by sexual orientation. Bisexual participants were significantly younger than other participants, followed by lesbian/gay participants, with no differences in age between heterosexual and prefer-not-to-say participants. Participants who self-identified as lesbian/gay were less likely to be female and more likely to be white, and prefer-not-to-say participants less likely to be white than other participants. Heterosexual participants were least likely and bisexual participants were most likely to be single. Bisexual and prefer-not-to-say participants were more likely to be from lower social grades, and lesbian/gay participants were least likely to be from lower social grades. Lesbian/gay participants were also most likely to have post-16 qualification and bisexuals were more likely than all other groups to report a disability. There were some small regional differences, with lesbian/gay participants being most likely to reside in South England and prefer-not-to-say participants most likely to live in Central England. All groups differed in terms of internet access, with more than daily use being most common among lesbian/gay participants, followed by bisexual and heterosexual participants and being least common among prefer-not-to-say participants.

What is the prevalence of tobacco and hazardous alcohol use in a large LGB population sample? Does it differ by gender or from the heterosexual population?

Table 1 provides prevalence estimates of sexual orientation identity in this sample. As there was a significant interaction between sexual orientation and gender for hazardous alcohol use (Wald $X^2(3)=15.86$, $p=0.001$) and a near significant interaction for tobacco use (Wald $X^2(3)=7.51$, $p=0.057$), further analysis on health behaviours was stratified by gender (see Tables 3 and 4).

Differences in tobacco and hazardous alcohol use as a function of sexual orientation identities were more pronounced among women than men (Figure 1A and 1B). Irrespective of gender, tobacco use was most prevalent among bisexual participants who were nearly twice as likely to smoke as their heterosexual counterparts (for men: 30.7%, 95%CI 23.7%-30.7% vs 20.4%, 95%CI 19.9%-21.0%; OR 1.94, 95%CI 1.36-2.77; for women: 32.4%, 95%CI 25.9%-39.6% vs 17.5%, 95%CI 17.0%-18.0%; OR 2.25, 95%CI 1.65-3.08). Similarly, those who self-identified as lesbian/gay were also more likely to smoke than

For peer review only - http://bmjopen.bmj.com/site/about/guidelines.xhtml

Table 2: Sociodemographic characteristics of sample by sexual orientation identity

Characteristic	Total (n=44,030)	Heterosexual (n=41,058)	Bisexual (n=316)	Lesbian/Gay (n=498)	Prefer-not-to-say (n=2,158)	P-value
Mean (SD) Age (years)	47.1 (18.6)	47.3 (18.6)[a]	32.5 (14.6)[b]	39.0 (15.4)[c]	47.6 (19.1)[a]	<0.001
% (N) Women	50.9 (22,406)	51.1 (20,962)[a]	55.7 (176)[a]	37.1 (185)[b]	50.2 (1,083)[a]	<0.001
% (N) White	86.7 (38,192)	87.0 (35,713)[a]	87.4 (277)[a]	96.0 (478)[b]	79.9 (1,724)[c]	<0.001
% (N) Single	41.4 (18,244)	40.4 (16,600)[a]	64.4 (204)[b]	55.6 (277)[b,c]	53.9 (1,163)[c]	<0.001
% (N) Social grade C2DE	45.4 (19.971)	45.1 (18,533)[a]	52.7 (167)[b]	33.4 (166)[c]	51.2 (1,105)[b]	<0.001
% (N) No post-16 qualification	34.3 (15,082)	34.5 (14,156)[a]	28.4 (90)[a]	19.5 (97)[b]	34.2 (739)[a]	<0.001
% (N) With disability	10.3 (4,541)	10.2 (4,188)[a]	19.9 (63)[b]	13.5 (67)[a]	10.3 (223)[a]	<0.001
% (N) Region		[a]	[a]	[b]	[c]	<0.001
North	28.8 (12,665)	29.3 (12,043)	33.4 (106)	32.5 (162)	16.4 (354)	
Central	30.0 (13,209)	29.8 (12,243)	31.5 (100)	18.9 (94)	35.8 (772)	
South	41.2 (18,158)	40.9 (16,773)	35.0 (110)	48.6 (242)	47.8 (1,032)	
% (N) Internet access		[a]	[b]	[c]	[d]	<0.001
Never	12.8 (5,651)	12.7 (5,231)	9.5 (30)	3.4 (17)	17.3 (373)	
≤ Daily	19.8 (8,718)	20.0 (8,218)	12.0 (38)	11.0 (55)	18.9 (407)	
> Daily	67.4 (29,661)	67.2 (27,609)	78.5 (248)	85.5 (426)	63.9 (1,378)	

[a,b,c,d]Different letters for groups in each row indicate significant differences between these groups after controlling for multiple comparisons ($p<0.05$), same letters indicate no group differences; please note that weighted data are shown

For peer review only

those with a heterosexual identity (for men: 25.9%, 95%CI 21.3%-31.0%; OR 1.63, 95%CI 1.16-2.30; for women: 24.9%, 95%CI 19.2%-32.6%; OR 1.48, 95%CI 1.14-1.91). However, while for male prefer-not-to-say participants tobacco use prevalence was similar to heterosexual participants (22.7%, 95%CI 20.3%-25.3%; OR 1.11, 95%CI 0.96-1.28), it was significantly lower for female prefer-not-to-say participants (15.5%, 95%CI 13.5%-17.8%; OR 0.82, 95%CI 0.69-0.97; Figure 1A).

The pattern for hazardous alcohol across sexual orientation identities also differed between men and women (Figure 1B). Irrespective of gender, prefer-not-to-say participants had the lowest prevalence of hazardous alcohol use, at nearly half the prevalence observed among heterosexual participants (for men: 13.7%; 95%CI 11.8-16.0% vs 18.4%, 95%CI 17.9%-18.9%; OR 0.68, 95%CI 0.57-0.82; for women: 4.1%, 95%CI 3.0%-5.4% vs 8.3%, 95%CI 7.9%-8.7%; OR 0.44, 95%CI 0.32-0.61). However, while for men hazardous alcohol use was greatest among those who self-identified as gay, at more than twice the prevalence of heterosexual men (30.0%, 25.2%-35.3%; OR 2.23, 95%CI 1.74-2.85), followed by bisexual men (24.3%, 95%CI 17.9%-32.1%; OR 1.64, 95%CI 1.12-2.41), a different pattern was observed in women. Bisexual women had the highest prevalence rates, being more than three times as likely as heterosexual women to engage in hazardous alcohol use (24.4%, 95%CI 18.7%-31.3%; OR 3.75, 95%CI 2.67-5.28), followed by lesbian participants (19.0%, 95%CI 14.0%-25.3%; OR 2.53, 95%CI 1.71-3.75).

Tables 3A and 3B provide information about tobacco use and hazardous alcohol use characteristics by sexual orientation identity in women and men. Among current or recent tobacco users, bisexual women appeared less dependent than heterosexual women (as measured by the heaviness of smoking index), were more likely to smoke roll-your-own cigarettes and consequently spent less money on tobacco than other groups (Table 3A). There were no group differences in hazardous alcohol use characteristics among women. By contrast, among men, there were notable differences between sexual orientation identities in hazardous alcohol use but not tobacco use characteristics (Table 3B). Hazardous drinkers in the prefer-not-to-say group reported greater urges to drink, and gay men reported greater motivation to cut-down on alcohol and had made more attempts to cut-down in the last year compared with heterosexual men.

For peer review only - http://bmjopen.bmj.com/site/about/guidelines.xhtml

Table 3A: Tobacco and hazardous alcohol use characteristics by sexual orientation identity in women

	Total	Heterosexual	Bisexual	Lesbian	Prefer-not-to-say	P-value
Tobacco users*	(n=4,243)	(n=3,952)	(n=60)	(n=50)	(n=181)	
Mean (SD) Cigarettes per day[†]	10.8 (7.7)	10.8 (7.6)	8.1 (7.8)	10.8 (10.3)	10.8 (8.5)	0.091
% (N) Primarily RYO use[†]	43.1 (1,532)	42.4 (1,406)[a]	65.3 (32)[b]	50.0 (20)[a,b]	48.1 (74)[a,b]	0.005
Mean (SD) HSI	1.8 (1.5)	1.8 (1.5)[a]	1.2 (1.4)[b]	1.6 (1.9)[a,b]	1.7 (1.4)[a,b]	0.011
Mean (SD) Urge to smoke	2.9 (1.2)	2.9 (1.2)	2.6 (1.1)	2.8 (1.3)	2.7 (1.1)	0.036
Mean (SD) MTSS[‡]	3.3 (2.0)	3.3 (2.0)	3.3 (2.0)	3.6 (2.0)	3.2 (2.0)	0.775
Mean (SD) Spent per week (£)[‡]	22.5 (19.4)	22.7 (19.4)[a]	14.4 (12.9)[b]	26.5 (27.9)[a]	20.4 (17.0)[a,b]	0.003
% (N) Quit attempt last year	36.0 (1,489)	36.3 (1,397)	31.7 (19)	40.0 (20)	29.9 (53)	0.281
% (N) GP advice	39.9 (1,691)	40.2 (1,587)	25.4 (15)	32.0 (16)	40.3 (73)	0.087
Hazardous alcohol users[§]	(n=4,351)	(n=4,100)	(n=59)	(n=74)	(n=118)	
Mean (SD) Urge to drink	1.5 (0.9)	1.5 (0.9)	1.6 (1.2)	1.8 (1.2)	1.7 (1.0)	0.011
Mean (SD) Motivation to cut down drinking	1.9 (1.6)	1.8 (1.6)	1.9 (1.4)	2.1 (1.9)	2.1 (1.9)	0.184
Mean (SD) Spent per week (£)	14.7 (14.1)	14.6 (14.0)	16.5 (16.6)	19.0 (16.5)	14.8 (11.4)	0.050
% (N) Attempt to cut-down last year	16.3 (676)	16.1 (629)	16.1 (9)	20.3 (15)	21.7 (23)	0.355
% (N) GP advice	7.3 (318)	7.3 (301)	10.2 (6)	6.8 (5)	5.0 (6)	0.647

*Current or past year tobacco users; [†]Current or past year cigarette smokers only; [‡]Current tobacco users only; [§]AUDIT score≥8 or AUDIT-C≥5 score RYO – roll-your-own; HSI – Heaviness of smoking index; MTSS-Motivation to Stop Scale; [a,b,c,d]Different letters for groups in each row indicate significant differences between these groups after controlling for multiple comparisons ($p<0.05$), same letters indicate no group differences; please note that weighted data are shown

For peer review only - http://bmjopen.bmj.com/site/about/guidelines.xhtml

Table 3B: Tobacco and hazardous alcohol use characteristics by sexual orientation identity in men

	Total	Heterosexual	Bisexual	Gay	Prefer-not-to-say	P-value
Tobacco users*	(n=4,834)	(n=4,426)	(n=51)	(n=87)	(n=269)	
Mean (SD) Cigarettes per day†	11.8 (8.7)	11.8 (8.6)	14.3 (17.9)	11.5 (10.1)	11.5 (8.1)	0.256
% (N) Primarily RYO use†	51.0 (2,034)	50.7 (1,858)	60.0 (21)	42.7 (32)	58.0 (123)	0.057
Mean (SD) HSI	1.8 (1.5)	1.8 (1.5)	2.1 (1.8)	1.9 (1.7)	1.9 (1.5)	0.670
Mean (SD) Urge to smoke	2.8 (1.2)	2.8 (1.2)	2.6 (1.2)	3.0 (1.1)	2.9 (1.2)	0.103
Mean (SD) MTSS‡	3.1 (1.9)	3.1 (1.9)	3.2 (2.2)	3.1 (2.0)	2.9 (1.9)	0.344
Mean (SD) Spent per week (£)‡	23.0 (21.4)	23.2 (21.6)	20.3 (18.4)	21.9 (17.7)	21.0 (20.0)	0.350
% (N) Quit attempt last year	32.1 (1,508)	32.1 (1,380)	41.2 (21)	39.5 (34)	28.2 (73)	0.115
% (N) GP advice	33.1 (1,600)	32.8 (1,450)	48.1 (25)	32.2 (28)	36.1 (97)	0.086
Hazardous alcohol users§	(n=7,768)	(n=7,285)	(n=58)	(n=154)	(n=272)	
Mean (SD) Urge to drink	1.7 (1.0)	1.7 (1.0)[a]	2.0 (1.3)[a,b]	1.7 (1.2)[a,b]	2.0 (1.0)[b]	<0.001
Mean (SD) Motivation to cut down drinking	1.7 (1.4)	1.7 (1.4)[a]	1.9 (1.7)[a,b]	2.1 (1.7)[b]	1.7 (1.4)[a,b]	0.005
Mean (SD) Spent per week (£)	21.8 (20.2)	21.6 (20.2)	21.3 (21.5)	25.4 (23.0)	24.2 (17.1)	0.027
% (N) Attempt to cut-down last year	14.5 (1,086)	14.5 (1,009)[a]	10.5 (6)[a,b]	23.3 (35)[b]	14.5 (36)[a,b]	0.019
% (N) GP advice	9.9 (766)	10.1 (734)	8.6 (5)	8.4 (13)	5.2 (14)	0.056

*Current or past year tobacco users; †Current or past year cigarette smokers only; ‡Current tobacco users only; §AUDIT score≥8 or AUDIT-C≥5 score
RYO – roll-your-own; HSI – Heaviness of smoking index; MTSS-Motivation to Stop Scale; [a,b,c,d]Different letters for groups in each row indicate significant differences between these groups after controlling for multiple comparisons ($p<0.05$), same letters indicate no group differences; please note that weighted data are shown

For peer review only

For peer review only - http://bmjopen.bmj.com/site/about/guidelines.xhtml

Are differences in tobacco and hazardous alcohol use, if any, between the LGB and heterosexual population attenuated when controlling for other covariates?

The associations of sexual orientation identity with tobacco and hazardous alcohol use were re-examined in women and men, controlling for sociodemographic variables (Tables 4A and 4B). Younger age, being white, single, from a lower SES group, lacking post-16 qualifications, reporting a disability and living in Northern England were all independently associated with current tobacco use as was, in men only, lack of internet access. The association between sexual orientation with smoking status was removed in both women and men after including SES status, ethnicity, age, educational attainment and disability (and for women only: marital status) in the model. This was confirmed by both forward and backward logistic regressions.

By contrast, the association of sexual orientation identity with hazardous alcohol use persisted in both women and men, even after controlling for socio-demographic characteristics (Table 4). Compared with heterosexual women, women who self-identified as bisexual (OR 1.93; 95%CI 1.34-2.79) or lesbian (OR 1.60; 95%CI 1.06-2.40) were more likely to engage in hazardous alcohol use in adjusted analysis, while prefer-not-to-say women were less likely to do so (OR 0.57; 95%CI 0.41-0.79; Table 4A). Among men, those who self-identified as gay were more likely (OR 1.36; 95%CI 1.05-1.76) and prefer-not-to-say men were less likely (OR 0.79; 95%CI 0.65-0.96) than heterosexual men to engage in hazardous drinking. However, bisexual men no longer differed from heterosexual men in hazardous drinking after adjusting for sociodemographic covariates (OR 1.17, 95%CI 0.78-1.76; Table 4B). Being younger, white, single, from a higher SES group and having post-16 qualifications as well as accessing the internet and being from Northern England were all independently associated with hazardous drinking in both men and women.

For peer review only - http://bmjopen.bmj.com/site/about/guidelines.xhtml

Table 4A: Factors associated with tobacco and hazardous alcohol use in women

Factor	Tobacco use				Hazardous alcohol use			
	OR (95%CI)	P-value	aOR (95%CI)	P-value	OR (95%CI)	P-value	aOR (95%CI)	P-value
Age	0.98 (0.98-0.98)	<0.001	0.97 (0.97-0.98)	<0.001	0.97 (0.96-0.97)	<0.001	0.97 (0.97-0.97)	<0.001
White	3.56 (3.08-4.12)	<0.001	4.82 (4.15-5.61)	<0.001	4.45 (3.49-5.66)	<0.001	5.55 (4.34-7.12)	<0.001
Single	1.65 (1.54-1.77)	<0.001	1.46 (1.35-1.57)	<0.001	1.61 (1.45-1.77)	<0.001	1.58 (1.42-1.76)	<0.001
Social grade C2DE	2.33 (2.17-2.50)	<0.001	1.92 (1.77-2.08)	<0.001	0.76 (0.69-0.84)	<0.001	0.80 (0.71-0.89)	<0.001
No post-16 qualification	1.69 (1.58-1.81)	<0.001	1.58 (1.45-1.71)	<0.001	0.55 (0.49-0.61)	<0.001	0.78 (0.69-0.88)	<0.001
With disability	1.54 (1.40-1.70)	<0.001	1.50 (1.35-1.67)	<0.001	0.83 (0.71-0.97)	0.020	1.15 (0.97-1.36)	0.116
Region								
North (Ref)	1	<0.001	1	<0.001	1	<0.001	1	<0.001
Central	0.71 (0.65-0.77)		0.79 (0.72-0.87)		0.34 (0.30-0.39)		0.41 (0.36-0.47)	
South	0.62 (0.57-0.67)		0.83 (0.76-0.90)		0.40 (0.36-0.45)		0.51 (0.45-0.57)	
Internet access								
Never (Ref)	1	0.088	1	0.172	1	<0.001	1	<0.001
≤ Daily	0.94 (0.84-1.06)		0.97 (0.85-1.10)		2.05 (1.58-2.66)		1.39 (1.06-1.83)	
> Daily	1.04 (0.94-1.14)		0.90 (0.79-1.02)		5.11 (4.07-6.43)		2.25 (1.74-2.91)	
Sexual orientation identity								
Heterosexual (Ref)	1		1		1		1	
Bisexual	2.25 (1.65-3.08)	<0.001	1.38 (0.99-1.93)	0.076	3.75 (2.67-5.28)	<0.001	1.93 (1.34-2.79)	<0.001
Lesbian	1.63 (1.16-2.30)		1.34 (0.93-1.92)		2.53 (1.71-3.75)		1.60 (1.06-2.40)	
Prefer-not-to-say	0.82 (0.69-0.97)		0.92 (0.77-1.10)		0.44 (0.32-0.61)		0.57 (0.41-0.79)	

OR-odds ratio, aOR – adjusted odds ratio

For peer review only - http://bmjopen.bmj.com/site/about/guidelines.xhtml

For peer review only

Table 4B: Factors associated with tobacco and hazardous alcohol use in men

Factor	Tobacco use				Hazardous alcohol use			
	OR (95%CI)	P-value	aOR (95%CI)	P-value	OR (95%CI)	P-value	aOR (95%CI)	P-value
Age	0.98 (0.98-0.98)	<0.001	0.97 (0.97-0.98)	<0.001	0.98 (0.98-0.98)	<0.001	0.98 (0.98-0.98)	<0.001
White	1.28 (1.12-1.40)	<0.001	1.71 (1.55-1.87)	<0.001	6.28 (5.35-7.38)	<0.001	7.62 (6.46-8.99)	<0.001
Single	1.85 (1.73-1.97)	<0.001	1.26 (1.18-1.36)	<0.001	1.52 (1.42-1.63)	<0.001	1.32 (1.22-1.43)	<0.001
Social grade C2DE	2.19 (2.05-2.34)	<0.001	1.74 (1.61-1.87)	<0.001	0.75 (0.70-0.80)	<0.001	0.81 (0.74-0.88)	<0.001
No post-16 qualification	1.73 (1.62-1.85)	<0.001	1.52 (1.42-1.64)	<0.001	0.70 (0.65-0.76)	<0.001	0.86 (0.79-0.94)	0.001
With disability	1.52 (1.38-1.67)	<0.001	1.53 (1.38-1.70)	<0.001	0.84 (0.75-0.94)	0.003	0.95 (0.84-1.08)	0.426
Region								
North (Ref)	1	<0.001	1	0.002	1	<0.001	1	<0.001
Central	0.82 (0.75-0.89)		0.86 (0.79-0.93)		0.37 (0.33-0.40)		0.44 (0.40-0.48)	
South	0.78 (0.72-0.84)		0.91 (0.84-0.99)		0.50 (0.47-0.55)		0.60 (0.56-0.66)	
Internet access								
Never (Ref)	1	<0.001	1	<0.001	1	<0.001	1	<0.001
≤ Daily	0.80 (0.72-0.89)		0.77 (0.68-0.86)		1.42 (1.23-1.64)		1.06 (0.91-1.24)	
> Daily	0.82 (0.75-0.90)		0.67 (0.60-0.75)		2.19 (1.94-2.47)		1.32 (1.14-1.52)	
Sexual orientation identity								
Heterosexual (Ref)	1		1		1		1	
Bisexual	1.94 (1.36-2.77)	<0.001	1.35 (0.93-1.95)	0.177	1.64 (1.12-2.41)	<0.001	1.17 (0.78-1.76)	0.006
Gay	1.48 (1.14-1.91)		1.22 (0.93-1.60)		2.23 (1.74-2.85)		1.36 (1.05-1.76)	
Prefer-not-to-say	1.11 (0.96-1.28)		1.05 (0.90-1.23)		0.68 (0.57-0.82)		0.79 (0.65-0.96)	

OR-odds ratio, aOR – adjusted odds ratio

For peer review only

For peer review only - http://bmjopen.bmj.com/site/about/guidelines.xhtml

Discussion

Our study found that in England, LGB men and women appear to have higher rates of tobacco and hazardous alcohol use. This is in agreement with previous studies of young people and adults, reporting greater risk of sexual minorities engaging in tobacco and hazardous alcohol use than their heterosexual counterparts [8 30-32]. Those who elected not to disclose their sexual orientation were less likely to engage in these risky health behaviours. In general, it appears that differences were more pronounced among women than men, as has been previously reported [33]. However, contrary to previous work, we found that differences between sexual orientation identities in tobacco use disappeared when socio-demographic variables were taken into account. Differences in hazardous alcohol use for different sexual orientation identities were also attenuated, especially among men, but remained significant when controlling for these covariates.

Differences in tobacco use between LGB and heterosexual participants in this study appear to be explained in the most part by underlying differences in major socio-demographic characteristics. This finding suggests that UK tobacco control seems to have achieved largely equitable reductions in prevalence, irrespective of sexual orientation. However, it is important to remember that tobacco use disparities remain in this group [9 30 34], even if these may not be attributable to sexual orientation alone, and further work needs to be done to support minority groups to reduce tobacco consumption. By contrast, the association of hazardous alcohol use with being LGB persisted after controlling for major socio-demographic confounders. This persistence suggests that there may exist specific influences that put sexual minorities at greater risk of hazardous alcohol use and as such require targeted health promotion. Moreover, the importance of targeting health messages to lesbian/bisexual women differently to gay/bisexual men is underlined by the association of being from a sexual minority with both tobacco and hazardous alcohol use remained stronger for women in adjusted analysis [35]. This finding replicates existing evidence of higher risk in this specific group [13 32 36],

For peer review only - http://bmjopen.bmj.com/site/about/guidelines.xhtml

The finding that the association of sexual orientation identity with tobacco use was attenuated after controlling for socio-demographic characteristics could be due to several reasons. First, self-identification as LGB is much greater in younger than older age groups [22] as are smoking rates. Second, participants who identified as lesbian/gay were more likely to be male and white and LGB participants were more likely to be single, all factors associated with greater smoking rates in the general population [22 37]. Third, the results may suggest a genuine shift in the environment encountered by sexual minorities. The social-ecological model [38] posits that interactions with the environment determines risk behaviour. Therefore reduced differences in tobacco use could indicate that the environment for sexual minorities and the majority is becoming increasingly similar. Moreover, the introduction of 'smokefree' laws may have had a disproportionately greater impact on tobacco use among sexual minorities as they are more likely to spend time in social, recreational spaces [16] in which smoking used to be the norm. It is more difficult to explain the role of deprivation (as measured by education or socio-economic group). Lesbian/gay, though not bisexual, participants had both lower levels of deprivation and higher smoking rates, despite the fact that deprivation is strongly and positively associated with tobacco use [39]. Smoking as a health behaviour may be performed differently in these groups, both compared to each other, and in comparison to heterosexuals [7]. It is also possible that the association of deprivation with tobacco use may be less pronounced in younger age groups and in those who self-identify as LGB, and therefore deprivation may have a different relationship with tobacco use among sexual orientation minorities. This issue deserves further investigation.

The persistent association with hazardous alcohol use among the LGB participants in this study highlights the negative behavioural consequences experienced by social minorities, not easily explained by general socio-demographic covariates. One potential explanatory factor not explored in the current analysis is the increased risk of mental health problems in the LGB community [5] which is also associated with problem drinking [40]. Other factors include the use of recreational spaces by sexual minorities where alcohol drinking may be the norm. It is interesting to note that prefer-not-to-say participants were less likely to engage in hazardous alcohol use, whereas no differences were observed in relation to tobacco use. This may be explained in part by societal/religious stigma imposed on people, i.e. many religions that adapt a

For peer review only - http://bmjopen.bmj.com/site/about/guidelines.xhtml

negative stance towards sexual minorities and lead to non-disclosure of sexual minority identity [41], often also have punitive views on alcohol resulting in lower consumption [42]. The finding that the association with hazardous alcohol use is particularly strong among sexual minority women has been previously reported [10 43]. This may be an expression of gender non-conformity (i.e. to go against stereotypical views of female vs. male drinking behaviour [44]) and also reflect the fact that women are more likely to experience double discrimination [45] which may increase the propensity to engage in risky health behaviours as a coping mechanism.

This study had a number of limitations. The cross-sectional nature of the design makes it difficult to assess causal pathways. We did attempt to reduce the risk of unmeasured bias by controlling for a range of known socio-demographic confounders for tobacco and hazardous alcohol use. Moreover, while the subgroup of participants with a minority sexual orientation identity was relatively small, this sample was representative of the general population of England. As has been previously discussed, sexuality can be measured in different ways. Here a measure of sexual orientation identity was used, as this is argued to be the most relevant dimension to investigate the relationship of sexual orientation with disadvantage [46]. Future research should consider investigating this question using measures of behaviour and attraction: groups who do not choose to identify as LGB but carry out same-sex behaviour for instance, may be quite different. Not everyone is comfortable adopting an identity label and some may have not wanted to select one of the options offered by the ONS question. Lastly, no objective measure of tobacco and alcohol use was taken; however, both behaviours were assessed using validated and reliable scales and low demand, anonymous studies tend to provide relatively unbiased results [47].

In conclusion, sexual orientation disparities in tobacco and hazardous alcohol use persist in England, with LGB people exhibiting greater levels of risky health behaviours. However, differences in tobacco but not hazardous alcohol use appear mainly associated with general socio-demographic differences between LGB and heterosexual groups. Further research is now needed to consider the explanatory factors and to develop interventions to remove health inequalities in these populations.

For peer review only - http://bmjopen.bmj.com/site/about/guidelines.xhtml

References

1. Dilley JA, Simmons KW, Boysun MJ, Pizacani BA, Stark MJ. Demonstrating the importance and feasibility of including sexual orientation in public health surveys: health disparities in the Pacific Northwest. Am J Public Health 2010;**100**(3):460-7 doi: 10.2105/AJPH.2007.130336[published Online First: Epub Date]|.

2. McCabe SE, Hughes TL, Bostwick W, Morales M, Boyd CJ. Measurement of sexual identity in surveys: implications for substance abuse research. Arch Sex Behav 2012;**41**(3):649-57 doi: 10.1007/s10508-011-9768-7[published Online First: Epub Date]|.

3. Haseldon L, Joloza T. *Measuring Sexual Identity: A Guide for Researchers*. Newport, Wales: Office for National Statistics, 2009.

4. Laumann EO, Gagnon JH, Michael RT, Michaels S. *The social organization of sexuality: Sexual practices in the United States*. Chicago: University of Chicago Press, 1994.

5. Semlyen J, King M, Varney J, Hagger-Johnson G. Sexual orientation and symptoms of common mental disorder or low wellbeing: combined meta-analysis of 12 UK population health surveys. BMC Psychiatry 2016;**16**(1):67 doi: 10.1186/s12888-016-0767-z[published Online First: Epub Date]|.

6. Cochran SD. Emerging issues in research on lesbians' and gay men's mental health: does sexual orientation really matter? Am Psychol 2001;**56**(11):931-47

7. Boehmer U, Miao X, Linkletter C, Clark MA. Adult health behaviors over the life course by sexual orientation. Am J Public Health 2012;**102**(2):292-300 doi: 10.2105/AJPH.2011.300334[published Online First: Epub Date]|.

8. Rosario M, Corliss HL, Everett BG, et al. Sexual orientation disparities in cancer-related risk behaviors of tobacco, alcohol, sexual behaviors, and diet and physical activity: pooled Youth Risk Behavior Surveys. Am J Public Health 2014;**104**(2):245-54 doi: 10.2105/AJPH.2013.301506[published Online First: Epub Date]|.

9. Blosnich J, Lee JG, Horn K. A systematic review of the aetiology of tobacco disparities for sexual minorities. Tob Control 2013;**22**(2):66-73 doi: 10.1136/tobaccocontrol-2011-050181[published Online First: Epub Date]|.

10. Bloomfield K, Wicki M, Wilsnack S, Hughes T, Gmel G. International differences in alcohol use according to sexual orientation. Subst Abus 2011;**32**(4):210-9 doi: 10.1080/08897077.2011.598404[published Online First: Epub Date]|.

11. Chakraborty A, McManus S, Brugha TS, Bebbington P, King M. Mental health of the non-heterosexual population of England. Br J Psychiatry 2011;**198**(2):143-8 doi: 10.1192/bjp.bp.110.082271[published Online First: Epub Date]|.

12. Collier KL, van Beusekom G, Bos HM, Sandfort TG. Sexual orientation and gender identity/expression related peer victimization in adolescence: a systematic review of associated psychosocial and health outcomes. J Sex Res 2013;**50**(3-4):299-317 doi: 10.1080/00224499.2012.750639[published Online First: Epub Date]|.

13. King M, Semlyen J, Tai SS, et al. A systematic review of mental disorder, suicide, and deliberate self harm in lesbian, gay and bisexual people. BMC Psychiatry 2008;**8**(1):70 doi: citeulike-article-id:8338079
doi: 10.1186/1471-244x-8-70[published Online First: Epub Date]|.

For peer review only - http://bmjopen.bmj.com/site/about/guidelines.xhtml

14. Hawkins JD, Catalano RF, Miller JY. Risk and protective factors for alcohol and other drug problems in adolescence and early adulthood: implications for substance abuse prevention. Psychol Bull 1992;**112**(1):64-105

15. Swendsen J, Conway KP, Degenhardt L, et al. Mental disorders as risk factors for substance use, abuse and dependence: results from the 10-year follow-up of the National Comorbidity Survey. Addiction 2010;**105**(6):1117-28 doi: 10.1111/j.1360-0443.2010.02902.x[published Online First: Epub Date]|.

16. Talley AE, Sher KJ, Littlefield AK. Sexual orientation and substance use trajectories in emerging adulthood. Addiction 2010;**105**(7):1235-45 doi: 10.1111/j.1360-0443.2010.02953.x[published Online First: Epub Date]|.

17. Talley AE, Sher KJ, Steinley D, Wood PK, Littlefield AK. Patterns of alcohol use and consequences among empirically derived sexual minority subgroups. J Stud Alcohol Drugs 2012;**73**(2):290-302

18. Aronsson H, Lind J, Ghirlanda S, Enquist M. Parental influences on sexual preferences: the case of attraction to smoking. J Evol Psychol 2011;**9** doi: http://dx.doi.org/10.1556/JEP.9.2011.12.1[published Online First: Epub Date]|.

19. Smith EA, Malone RE. The outing of Philip Morris: advertising tobacco to gay men. Am J Public Health 2003;**93**(6):988-93

20. Fidler JA, Shahab L, West O, et al. 'The Smoking Toolkit Study': A national study of smoking and smoking cessation in England. BMC Public Health 2011;**11**(1):479

21. Beard E, Brown J, West R, et al. Protocol for a national monthly survey of alcohol use in England with 6-month follow-up: 'the Alcohol Toolkit Study'. BMC Public Health 2015;**15**:230 doi: 10.1186/s12889-015-1542-7[published Online First: Epub Date]|.

22. Office for National Statistics. Integrated Household Survey, January to December 2014: Experimental Statistics. Secondary Integrated Household Survey, January to December 2014: Experimental Statistics 2015.

23. Heatherton TF, Kozlowski LT, Frecker RC, Rickert W, Robinson J. Measuring the heaviness of smoking: using self-reported time to the first cigarette of the day and number of cigarettes smoked per day. Br J Addict 1989;**84**(7):791-99

24. Fidler JA, Shahab L, West R. Strength of urges to smoke as a measure of severity of cigarette dependence: comparison with the Fagerstrom Test for Nicotine Dependence and its components. Addiction 2011;**106**(3):631-38

25. Kotz D, Brown J, West R. Predictive validity of the Motivation To Stop Scale (MTSS): A single-item measure of motivation to stop smoking. Drug Alcohol Depend. 2013;**128**(1-2):15-19

26. Babor T, HIggins-Biddle J, Saunders J, Monteiro M. *AUDIT: the Alcohol Use Disorders Identification Test: guidelines for use in primary care.* 2nd ed. Geneva, Switzerland: WHO, 2001.

27. National Readership Survey. http://www.nrs.co.uk/nrs-print/methodology/technical-information/ (accessed 23.01.15). 2015

28. Brown J, West R, Angus C, et al. Comparison of brief interventions in primary care on smoking and excessive alcohol consumption: a population survey in England. Br J Gen Pract 2016;**66**(642):e1-9 doi: 10.3399/bjgp16X683149[published Online First: Epub Date]|.

For peer review only - http://bmjopen.bmj.com/site/about/guidelines.xhtml

29. Benjamini Y, Hochberg Y. Controlling the False Discovery Rate - A Practical and Powerful Approach to Multiple Testing. J R Statist Soc, B 1995;**57**(1):289-300

30. Lee JG, Griffin GK, Melvin CL. Tobacco use among sexual minorities in the USA, 1987 to May 2007: a systematic review. Tob Control 2009;**18**(4):275-82 doi: 10.1136/tc.2008.028241[published Online First: Epub Date]|.

31. Ryan H, Wortley PM, Easton A, Pederson L, Greenwood G. Smoking among lesbians, gays, and bisexuals: a review of the literature. Am J Prev Med 2001;**21**(2):142-9

32. Hagger-Johnson G, Taibjee R, Semlyen J, et al. Sexual orientation identity in relation to smoking history and alcohol use at age 18/19: cross-sectional associations from the Longitudinal Study of Young People in England (LSYPE). BMJ Open 2013;**3**(8):e002810 doi: 10.1136/bmjopen-2013-002810[published Online First: Epub Date]|.

33. McCabe SE, Hughes TL, Bostwick WB, West BT, Boyd CJ. Sexual orientation, substance use behaviors and substance dependence in the United States. Addiction 2009;**104**(8):1333-45 doi: 10.1111/j.1360-0443.2009.02596.x[published Online First: Epub Date]|.

34. Johnson SE, Holder-Hayes E, Tessman GK, King BA, Alexander T, Zhao X. Tobacco Product Use Among Sexual Minority Adults: Findings From the 2012-2013 National Adult Tobacco Survey. Am J Prev Med 2016;**50**(4):e91-e100 doi: 10.1016/j.amepre.2015.07.041[published Online First: Epub Date]|.

35. Fallin A, Goodin A, Lee YO, Bennett K. Smoking characteristics among lesbian, gay, and bisexual adults. Prev Med 2015;**74**:123-30 doi: 10.1016/j.ypmed.2014.11.026[published Online First: Epub Date]|.

36. Branstrom R, Hatzenbuehler ML, Pachankis JE. Sexual orientation disparities in physical health: age and gender effects in a population-based study. Soc Psychiatry Psychiatr Epidemiol 2016;**51**(2):289-301 doi: 10.1007/s00127-015-1116-0[published Online First: Epub Date]|.

37. Office for National Statistics. Adult Smoking Habits in Great Britain, 2013. Secondary Adult Smoking Habits in Great Britain, 2013 2015.

38. Bronfenbrenner U. *The ecology of human development: Experiments by nature and design*. Cambridge, MA: Havard University Press, 1979.

39. Jarvis MJ, Wardle J. Social patterning of individual health behaviours: the case of cigarette smoking. In: Marmot M, Wilkinson RG, eds. Social Determinants of Health. New York: Oxford University Press, 2005:224-37.

40. Makela P, Raitasalo K, Wahlbeck K. Mental health and alcohol use: a cross-sectional study of the Finnish general population. Eur J Public Health 2015;**25**(2):225-31 doi: 10.1093/eurpub/cku133[published Online First: Epub Date]|.

41. Schrimshaw EW, Downing MJ, Jr., Cohn DJ. Reasons for Non-Disclosure of Sexual Orientation Among Behaviorally Bisexual Men: Non-Disclosure as Stigma Management. Arch Sex Behav 2016 doi: 10.1007/s10508-016-0762-y[published Online First: Epub Date]|.

42. Michalak L, Trocki K, Bond J. Religion and alcohol in the U.S. National Alcohol Survey: how important is religion for abstention and drinking? Drug Alcohol Depend 2007;**87**(2-3):268-80 doi: 10.1016/j.drugalcdep.2006.07.013[published Online First: Epub Date]|.

For peer review only - http://bmjopen.bmj.com/site/about/guidelines.xhtml

43. Midanik L, Drabble L, Trocki K, Sell R. Sexual orientation and alcohol use: identiy versus behavior measures. Journal of LGBT Research 2006;**3**:25-35 doi: 10.1300/J463v03n01_04[published Online First: Epub Date]|.

44. Rosario M, Schrimshaw EW, Hunter J. Butch/Femme differences in substance use and abuse among young lesbian and bisexual women: examination and potential explanations. Subst Use Misuse 2008;**43**(8-9):1002-15 doi: 10.1080/10826080801914402[published Online First: Epub Date]|.

45. Bowleg L, Huang J, Brooks K, Black A, Burkholder G. Triple jeopardy and beyond: mulitple minority stress and resilience among black lesbians. Journal of Lesbian Studies 2003;**7**:87-108

46. Vizard T. Measuring sexual identity in the United Kingdom. Journal of Bisexuality 2014;**14**:524-43 doi: 10.1080/15299716.2914.9311830[published Online First: Epub Date]|.

47. Bonevski B, Campbell E, Sanson-Fisher RW. The validity and reliability of an interactive computer tobacco and alcohol use survey in general practice. Addict Behav 2010;**35**(5):492-8 doi: 10.1016/j.addbeh.2009.12.030[published Online First: Epub Date]|.

For peer review only

Acknowledgement

None.

Author contributions statement

CM, JB & RW conceived the original idea for this study. CM obtained funding. JB and RW managed the day-to-day running of the study. LS undertook the data analyses and wrote the initial draft with further input from all authors. LS is guarantor for this article. LS, JB, GHJ, SM, JS, RW and CM read, reviewed and approved the final version. All researchers listed as authors are independent from the funders and all final decisions about the research were taken without constraint by the investigators. LS had full access to all the data in the study and had final responsibility for the decision to submit for publication.

Additional Information

Funding

We are grateful to Cancer Research UK who funded this analysis (C52095/A18725). The Smoking Toolkit Study is primarily funded by Cancer Research UK (C1417/A14135; C36048/A11654; C44576/A19501) and has previously also been funded by Pfizer, GSK, and the Department of Health. The National Institute for Health Research (NIHR) School for Public Health Research funded data collection for the Alcohol Toolkit Study (SPHR-SWP-ALC-WP5). JB's post is funded by a fellowship from the Society for the Study of Addiction and CRUK also provide support (C1417/A14135); Robert West is funded by Cancer Research UK (C1417/A14135). SPHR is a partnership between the Universities of Sheffield; Bristol; Cambridge; Exeter; UCL; The London School for Hygiene and Tropical Medicine; the LiLaC collaboration between the Universities of Liverpool and Lancaster and Fuse; The Centre for Translational Research in Public Health, a collaboration between Newcastle, Durham, Northumbria, Sunderland and Teesside Universities. The views expressed are those of the authors(s) and not necessarily those of the NHS, NIHR, or Department of Health.

For peer review only

Conflict of Interest

LS has received a research grant and honoraria for a talk and travel expenses from a Pfizer, manufacturer of smoking cessation medications. JB has received unrestricted research funding from Pfizer to study smoking cessation. RW has received travel funds and hospitality from, and undertaken research and consultancy for, pharmaceutical companies that manufacture or research products aimed at helping smokers to stop. GHJ receives royalties from ‘'Introduction to Research Methods and Data Analysis in Psychology'’ (2013) and ‘Psychometric Assessment, Statistics and Report Writing’ (2013), both published by Pearson and ‘Introduction to Research Methods and Data Analysis in the Health Sciences’. He has also worked as a consultant for various universities and Public Health England. His substantive contribution to the manuscript was prior to June 2016. CM, SM and JS have no competing interests.

Data sharing

Headline data from the Toolkit Study are published monthly at http://www.smokinginengland.info/. Data will be made available upon request.

For peer review only

Figure legend

Figure 1: PNTS – Prefer-not-to-say; Error bars are 95% confidence intervals; All data are unadjusted

For peer review only

For peer review only - http://bmjopen.bmj.com/site/about/guidelines.xhtml

Figure 1: Prevalence of (A) tobacco use and (B) hazardous alcohol use by gender and sexual orientation identity

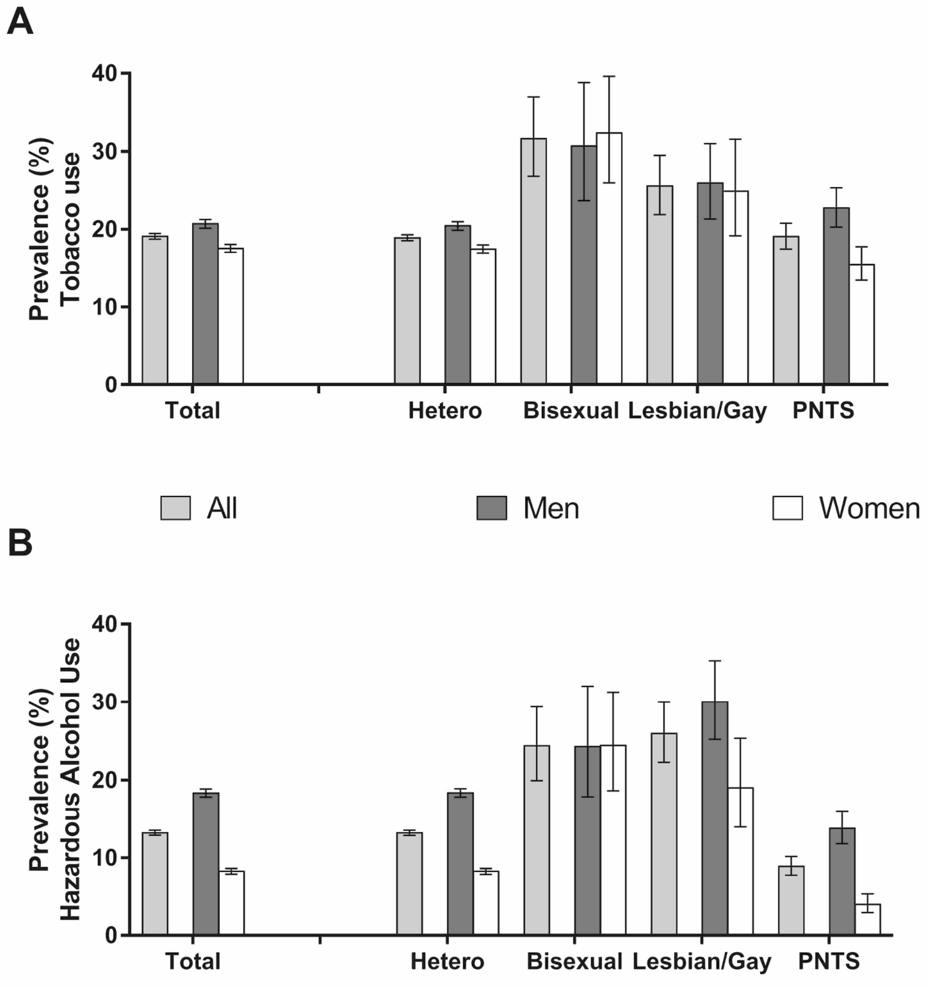

218x257mm (300 x 300 DPI)

STROBE Statement—checklist of items that should be included in reports of observational studies

	Item No	Recommendation
**Title and abstract**	1	(*a*) Indicate the study's design with a commonly used term in the title or the abstract **Page 1**
		(*b*) Provide in the abstract an informative and balanced summary of what was done and what was found **Page 2**
**Introduction**		
Background/rationale	2	Explain the scientific background and rationale for the investigation being reported **Page 4-5**
Objectives	3	State specific objectives, including any prespecified hypotheses **Page 5**
**Methods**		
Study design	4	Present key elements of study design early in the paper **Page 6**
Setting	5	Describe the setting, locations, and relevant dates, including periods of recruitment, exposure, follow-up, and data collection **Page 6**
Participants	6	(*a*) *Cohort study*—Give the eligibility criteria, and the sources and methods of selection of participants. Describe methods of follow-up<br>*Case-control study*—Give the eligibility criteria, and the sources and methods of case ascertainment and control selection. Give the rationale for the choice of cases and controls<br>*Cross-sectional study*—Give the eligibility criteria, and the sources and methods of selection of participants **Page 6**
		(*b*) *Cohort study*—For matched studies, give matching criteria and number of exposed and unexposed<br>*Case-control study*—For matched studies, give matching criteria and the number of controls per case
Variables	7	Clearly define all outcomes, exposures, predictors, potential confounders, and effect modifiers. Give diagnostic criteria, if applicable **Page 6-8**
Data sources/ measurement	8*	For each variable of interest, give sources of data and details of methods of assessment (measurement). Describe comparability of assessment methods if there is more than one group **Page 6-8**
Bias	9	Describe any efforts to address potential sources of bias **Page 8**
Study size	10	Explain how the study size was arrived at **N/A**
Quantitative variables	11	Explain how quantitative variables were handled in the analyses. If applicable, describe which groupings were chosen and why **Page 8**
Statistical methods	12	(*a*) Describe all statistical methods, including those used to control for confounding **Page 8**
		(*b*) Describe any methods used to examine subgroups and interactions **Page 8**
		(*c*) Explain how missing data were addressed **Page 6**
		(*d*) *Cohort study*—If applicable, explain how loss to follow-up was addressed<br>*Case-control study*—If applicable, explain how matching of cases and controls was addressed<br>*Cross-sectional study*—If applicable, describe analytical methods taking account of sampling strategy **Page 8**
		(*e*) Describe any sensitivity analyses **N/A**

Continued on next page

For peer review only

Results		
Participants	13*	(a) Report numbers of individuals at each stage of study—eg numbers potentially eligible, examined for eligibility, confirmed eligible, included in the study, completing follow-up, and analysed **Page 6**
		(b) Give reasons for non-participation at each stage **Page 6**
		(c) Consider use of a flow diagram **N/A**
Descriptive data	14*	(a) Give characteristics of study participants (eg demographic, clinical, social) and information on exposures and potential confounders **Page 9-10**
		(b) Indicate number of participants with missing data for each variable of interest **N/A**
		(c) *Cohort study*—Summarise follow-up time (eg, average and total amount)
Outcome data	15*	*Cohort study*—Report numbers of outcome events or summary measures over time
		Case-control study—Report numbers in each exposure category, or summary measures of exposure
		Cross-sectional study—Report numbers of outcome events or summary measures **Page 9, 11**
Main results	16	(*a*) Give unadjusted estimates and, if applicable, confounder-adjusted estimates and their precision (eg, 95% confidence interval). Make clear which confounders were adjusted for and why they were included **Page 9,11, 14-16**
		(*b*) Report category boundaries when continuous variables were categorized **N/A**
		(*c*) If relevant, consider translating estimates of relative risk into absolute risk for a meaningful time period **N/A**
Other analyses	17	Report other analyses done—eg analyses of subgroups and interactions, and sensitivity analyses **Page 9-16**
**Discussion**		
Key results	18	Summarise key results with reference to study objectives **Page 17**
Limitations	19	Discuss limitations of the study, taking into account sources of potential bias or imprecision. Discuss both direction and magnitude of any potential bias **Page 19**
Interpretation	20	Give a cautious overall interpretation of results considering objectives, limitations, multiplicity of analyses, results from similar studies, and other relevant evidence **Page 17-19**
Generalisability	21	Discuss the generalisability (external validity) of the study results **Page 19**
**Other information**		
Funding	22	Give the source of funding and the role of the funders for the present study and, if applicable, for the original study on which the present article is based **Page 25**

*Give information separately for cases and controls in case-control studies and, if applicable, for exposed and unexposed groups in cohort and cross-sectional studies.

Note: An Explanation and Elaboration article discusses each checklist item and gives methodological background and published examples of transparent reporting. The STROBE checklist is best used in conjunction with this article (freely available on the Web sites of PLoS Medicine at http://www.plosmedicine.org/, Annals of Internal Medicine at http://www.annals.org/, and Epidemiology at http://www.epidem.com/). Information on the STROBE Initiative is available at www.strobe-statement.org.

For peer review only

For peer review only - http://bmjopen.bmj.com/site/about/guidelines.xhtml

Sexual orientation identity and tobacco and hazardous alcohol use: findings from a cross-sectional English population survey

Journal:	*BMJ Open*
Manuscript ID	bmjopen-2016-015058.R1
Article Type:	Research
Date Submitted by the Author:	09-Jun-2017
Complete List of Authors:	Shahab, Lion; UCL, Dept of Behavioural Science and Health<br>Brown, Jamie; University College London, Psychology & Language Sciences<br>Hagger-Johnson, Gareth; UCL, Farr Institute<br>Michie, Susan; University College London, Centre for Outcomes Research and Effectivenes<br>Semlyen, Joanna; University of East Anglia Faculty of Medicine and Health Sciences<br>West, Robert; University College London, Behavioural Science and Health<br>Meads, Catherine; Anglia Ruskin University - Cambridge Campus, Faculty of Health, Social Care and Education
**Primary Subject Heading**:	Smoking and tobacco
Secondary Subject Heading:	Addiction, Epidemiology, Public health
Keywords:	EPIDEMIOLOGY, Health policy < HEALTH SERVICES ADMINISTRATION & MANAGEMENT, Substance misuse < PSYCHIATRY, PUBLIC HEALTH

SCHOLARONE™ Manuscripts

Sexual orientation identity and tobacco and hazardous alcohol use: findings from a cross-sectional English population survey.

Lion Shahab[1*], Jamie Brown[1,2], Gareth Hagger-Johnson[3], Susan Michie[2], Joanna Semlyen[4], Robert West[1], Catherine Meads[5]

[1] Department of Behavioural Science and Health, University College London, London, UK

[2] Department of Clinical, Educational and Health Psychology, University College London, London, UK

[3] Administrative Data Research Centre for England (ADRC-E), Farr Institute, University College London, London, UK

[4] Norwich Medical School, University of East Anglia, Norwich, UK

[5] Faculty of Health, Social Care and Education, Anglia Ruskin University, Cambridge, UK

*Correspondence to: Dr Lion Shahab, Department of Epidemiology & Public Health, University College London, 1–19 Torrington Place, London, WC1E 6BT, UK; Phone: +44 207679 1805; Fax: +44 207813 2848; Email: lion.shahab@ucl.ac.uk

Abstract: 299/300

Manuscript: 3850/4000 (excluding abstract, figures, tables, legends and references)

Figures and tables: 5/5

References: 51

For peer review only

Abstract

Objectives: To assess the association between tobacco and hazardous alcohol use and sexual orientation and whether such an association could be explained by other socio-demographic characteristics.

Design: Cross-sectional household survey conducted in 2014-2016.

Setting: England, UK.

Participants: Representative English population sample (pooled N=43,866).

Main Outcomes: Sexual orientation identity (lesbian/gay, bisexual, heterosexual, prefer-not-to-say); current tobacco and hazardous alcohol use (defined as Alcohol Use Disorders Identification Test Score ≥ 8). All outcomes were self-reported.

Results: Due to interactions between sexual orientation and gender for substance use, analyses were stratified by gender. Tobacco use prevalence was significantly higher among lesbian/gay (women: 24.9%, 95% confidence interval (CI) 19.2%-32.6%; men: 25.9%, 95%CI 21.3%-31.0%) and bisexual participants (women: 32.4%, 95%CI 25.9%-39.6%; men: 30.7%, 95%CI 23.7%-30.7%) and significantly lower for prefer-not-to-say participants in women (15.5%, 95%CI 13.5%-17.8%) but not men (22.7%, 95%CI 20.3%-25.3%) compared with heterosexual participants (women: 17.5%, 95%CI 17.0%-18.0%; men: 20.4%, 95%CI 19.9%-21.0%; p<0.001 for omnibus test). Similarly, hazardous alcohol use was significantly more prevalent for lesbian/gay (women: 19.0%, 95%CI 14.0%-25.3%; men: 30.0%, 25.2%-35.3%) and bisexual participants (women: 24.4%, 95%CI 18.7%-31.3%; men: 24.3%, 95%CI 17.9%-32.1%) and lower for prefer-not-to-say participants (women: 4.1%, 95%CI 3.0%-5.4%; men: 13.7%; 95%CI 11.8-16.0%) compared with heterosexuals (women: 8.3%, 95%CI 7.9%-8.7%; men: 18.4%, 95%CI 17.9%-18.9%; p<0.001 for omnibus test). However, after adjusting for socio-demographic confounders, tobacco use was similar across all sexual orientation groups among both women and men. By contrast, sexual orientation differences in hazardous alcohol use remained even after adjustment among women but not for bisexual and gay men.

Conclusions: In England, higher rates of tobacco use among sexual minority men and women appear to be attributable to other socio-demographic factors. Higher rates of hazardous alcohol use among sexual

For peer review only - http://bmjopen.bmj.com/site/about/guidelines.xhtml

For peer review only

minority men may also be attributable to these factors, whereas this is not the case for sexual minority women.

Key words: Tobacco use, hazardous alcohol use, sexual orientation identity, LGB, epidemiology

For peer review only

For peer review only - http://bmjopen.bmj.com/site/about/guidelines.xhtml

Strengths and limitations of this study

- Most research assessing sexual orientation and substance use involves small convenience samples, and data from representative samples is scarce. This study is one of only very few studies to investigate this issue in a large representative population survey
- This study used validated measures of sexual orientation and health behaviours (tobacco and hazardous alcohol use) and controlled for a wide range of confounders to assess the association between them.
- As is the case for all cross-sectional analysis we cannot infer causality from this study, and the measure of sexual orientation may not have captured all dimensions of this construct.

For peer review only

For peer review only - http://bmjopen.bmj.com/site/about/guidelines.xhtml

Introduction

Knowledge about the health behaviours of sexual minority (lesbian, gay, and bisexual, (LGB)) groups is necessary for monitoring health inequalities, developing public health policies, allocating resources and targeting high-risk groups for interventions.[1, 2]

There is a validated measure of sexual orientation for LGB groups developed by the Office of National Statistics [3] but despite recent equality and diversity legislation and the inclusion of sexual orientation in the National Health Service (NHS) Equality Delivery System, sexual orientation identity is not regularly monitored and/or reported by NHS organisations or collected in epidemiological research. This may in part be due to the complex nature of sexual orientation, which can be defined and assessed along dimensions of behaviour, identity or attraction, or a combination of these.[4] The different dimensions of identity, behaviour and attraction do not map easily onto each, and even within these dimensions, not all possible manifestations have been explored.[5] Other methodological issues include the fact that, historically, sexual minorities were relatively more hidden due to stigmatisation, resulting in, small, non-representative samples being studied.[6] This also means that a substantial proportion of participants who do not identify as heterosexual many not endorse any other sexual orientation in surveys for various reasons, including the sensitive nature of the topic or conservative attitudes.[7]

Due to this lack of data, the evidence base on health inequalities experienced by sexual minorities is sparse and has only recently begun to be explored. In the UK, LGB sexual identity and behaviour has been assessed in the National Attitudes of Sexual Attitudes and Lifestyles (NATSAL; 1990–1991, 1999–2001, 2010–2012), National Statistics Opinions Survey (in 2008–2009), Office for National Statistics (ONS) Integrated Household Survey (from 2009), Longitudinal Study of Young People in England (from 2009), British Cohort Study (2012), Health Survey for England (from 2010), Scottish Health Survey (from 2008), English Longitudinal Study of Ageing (2012/13) and Understanding Society (from 2011/12). In addition, several major cohort studies in the USA have included questions on sexual orientation identity in recent years.[8, 9] Yet, due to the methodological difficulties discussed above, most evidence on health behaviours

For peer review only - http://bmjopen.bmj.com/site/about/guidelines.xhtml

so far has come from small convenience samples, suggesting that sexual minorities are more likely to smoke tobacco or engage in harmful alcohol use.[9] Representative population data across the entire adult age range, especially from non-US samples, are scarce.[5, 10, 11]

Several possible mechanisms have been proposed that connect sexual orientation identity to health behaviours such as smoking and alcohol use. The concept of minority stress is often invoked to explain how heterosexism and homophobia are internalised,[12] perhaps leading people to self-medicate for psychological distress with cigarettes or other substances such as alcohol. A recent review found support for this theory, particularly in explaining associations with victimisation and substance use.[13] Another factor could be lower levels of self-esteem, wellbeing and greater propensity to mental health problems in this population brought on by biases towards sexual minorities [5, 6, 14] which in themselves have been linked to tobacco and alcohol abuse.[15, 16] Alternatively, LGB young people, in particular, may be more likely to access adult venues and clubs associated with use of intoxicating substances, including alcohol and illegal drugs.[17] Due to a lack of social support at school, from peers and family, LGB young people may seek this support and socialise in recreational spaces where peer norms encourage engagement in risky health behaviours,[18] or these behaviours may be adopted in order to appear older to gain access to a venue or to fit in.[19] Substance use may be sexually arousing for some individuals and subcultures, particularly when seen to signify masculinity.[20] Other commentators have noted the role of industry in targeting this community.[21]

There is a clear need for population level studies to investigate the extent of differences in tobacco and alcohol use by sexual minorities. For instance, it is possible that apparent differences in prevalence may be an artefact of other socio-demographic differences associated with sexual minorities or the use of non-representative samples. Better describing of tobacco and alcohol use behaviour and identifying effective drivers for change will help reduce health inequalities for the LGB community. This study aimed to assess the prevalence and association of tobacco and hazardous alcohol use with sexual orientation identity. Specifically, the study addressed the following questions:

For peer review only - http://bmjopen.bmj.com/site/about/guidelines.xhtml

1) What is the prevalence of tobacco and hazardous alcohol use and distribution of associated use characteristics in a large LGB population sample? Do they differ by gender or from the heterosexual population?

2) Are differences in tobacco and hazardous alcohol use prevalence, if any, between the LGB and heterosexual population sample attenuated when controlling for other sociodemographic covariates?

For peer review only

For peer review only - http://bmjopen.bmj.com/site/about/guidelines.xhtml

Methods

Study design and participants

Data were collected using cross-sectional household surveys of representative samples of the population of adults in England. The surveys are part of the ongoing series of Smoking and Alcohol Toolkit Studies, designed to provide tracking information about smoking, alcohol consumption and related behaviours in England. Each month a new sample of approximately 1,700 adults aged ≥16 years complete a face-to-face computer-assisted survey with a trained interviewer (see [22, 23] for full details). Current data were collected from March 2014 until May 2016 where complete data on tobacco, alcohol use, sexual orientation and other baseline socio-demographic variables were included. A total of 45,423 adults were surveyed in this time period, of whom 43,886 had complete data. The study received ethical approval from the University College London Research Ethics Committee and was carried out in accordance with the ethical principles on human research, as set out in the Declaration of Helsinki.

Measures

Sexual Identity

Sexual orientation was assessed by asking respondents to self-identify as (a) Bisexual; (b) Gay man/homosexual; (c) Gay woman/lesbian; (d) Heterosexual/straight; (e) Prefer not to say. This measure deviates slightly from the recommended ONS item (which also provides an 'Other' option) [3] but produces similar prevalence rates compared with the latest ONS Integrated Household Survey [24] (see Table 1).

Table 1: Prevalence of self-perceived sexual orientation identity in the Smoking and Alcohol Toolkit Study 2014-2016 and ONS Integrated Household Survey, 2014

	Toolkit Study	ONS
	% (95% Confidence Interval)	
Heterosexual/Straight	93.3 (93.0-93.5)	92.8 (92.6-93.0)
Bisexual	0.7 (0.6-0.8)	0.5 (0.4-0.6)
Lesbian/Gay	1.1 (1.0-1.2)	1.1 (1.0-1.2)
PNTS	4.9 (4.7-5.1)	5.3 (5.2-5.4)
Other	-	0.3 (0.3-0.3)

PNTS – Prefer-not-to-say

For peer review only - http://bmjopen.bmj.com/site/about/guidelines.xhtml

Tobacco use

To assess tobacco use, participants were asked if they (a) smoked cigarettes (including hand-rolled) every day; (b) smoked cigarettes (including hand-rolled) but not every day; (c) did not smoke cigarettes at all but did smoke tobacco of some kind (e.g. pipe or cigar); (d) had stopped smoking completely in the last year; (e) had stopped smoking completely more than a year ago; or (f) had never been a smoker (i.e. smoked for a year or more). Current smokers were classified as answering 'yes' to (a) to (c), and ex-smokers, classified as answering 'yes' to (d) or (e). Those answering 'yes' to (f) were classified as never-smokers and together with ex-smokers also classified as non-smokers to indicate current tobacco use (yes/no). Additionally, current smokers were asked questions to determine nicotine dependence (measured by heaviness of smoking index, (HSI[25]), and strength of urges to smoke, (SUTS[26]) as well as motivation to quit (measured by the motivation to stop scale, (MTSS) [27]). Smokers also provided an estimate of money spent per week on tobacco products, whether they had made at least one serious quit attempt in the last year and whether they had been asked about their smoking, received advice and/or support to stop from their GP.

Alcohol use

Alcohol use was assessed with the well-established and reliable Alcohol Use Disorders Identification Test (AUDIT) questionnaire, with a cut-off ≥ 8 to indicate hazardous alcohol use (yes/no).[28] Participants who scored as hazardous drinkers were further asked about their urges to drink (adapted from the SUTS) and their motivation to cut down alcohol consumption (adapted from the MTSS). They were also asked how much money they spent per week on alcohol for their own consumption, whether they had made a serious attempt to cut down in the last year and whether they had been asked about their drinking, received advice and/or support to cut down from their GP.

Covariates

Standard socio-demographic characteristics assessed included age (in years), sex (male/female), ethnicity (white/non-white), marital status (married, civil partnership or living with partner; yes/no), socio-

For peer review only

economic status; (ABC1/C2DE), disability; (yes/no), education (post-16 (post high-school) qualification; yes/no), England region; (North/Central/South) and internet use; (>daily/≤daily/never).

Analysis

Data were analysed with IBM SPSS Statistics Version 24.0. All data were weighted using the rim (marginal) weighting technique to match an English population profile on the dimensions of age, social grade, region, tenure, ethnicity and working status within sex derived from the English 2011 census, ONS 2013 mid-year estimates and a random probability survey conducted in 2014 for the National Readership Survey.[29] Further details on the weighting procedure are reported elsewhere.[30] Simple associations between study groups and continuous and categorical socio-demographic, tobacco use and alcohol use characteristics were assessed with one-way ANOVAs and chi-square analysis, respectively. General linear models (GLM) with a log-binomial link were used to analyse the association of sexual orientation with the binary outcome variables (current tobacco use; hazardous alcohol consumption) and to calculate prevalence ratios. We included interaction terms for sexual orientation by gender in the GLM to determine if there were systematic differences. Additional stepwise forward and backward logistic regression models were run to determine the individual impact of covariates on the outcome variables, being ordered by the magnitude of changes in variance in the model when covariates are added or removed. Family-wise error rate was corrected using the false discovery rate [31] and multiple comparisons were controlled for using the Sidak correction in post-hoc analysis. Only complete cases were analysed and missing data excluded.

For peer review only - http://bmjopen.bmj.com/site/about/guidelines.xhtml

Results

Table 1 provides information about the prevalence of sexual orientation in this sample and Table 2 provides a breakdown of associated socio-demographic characteristics. Bisexual participants were significantly younger than other participants, followed by lesbian/gay participants, with no differences in age between heterosexual and prefer-not-to-say participants. Participants who self-identified as lesbian/gay were less likely to be female and more likely to be white, and prefer-not-to-say participants less likely to be white than other participants. Heterosexual participants were least likely and bisexual participants were most likely to be single. Bisexual and prefer-not-to-say participants were more likely to be from lower social grades, and lesbian/gay participants were least likely to be from lower social grades. Lesbian/gay participants were also most likely to have post-16 qualification and bisexuals were more likely than all other groups to report a disability. There were some small regional differences, with lesbian/gay participants being most likely to reside in South England and prefer-not-to-say participants most likely to live in Central England. All groups differed in terms of internet access, with more than daily use being most common among lesbian/gay participants, followed by bisexual and heterosexual participants and being least common among prefer-not-to-say participants.

What is the prevalence of tobacco and hazardous alcohol use and distribution of associated use characteristics in a large LGB population sample? Do they differ by gender or from the heterosexual population?

As there was a significant interaction between sexual orientation and gender for hazardous alcohol use (Wald $X^2(3)=21.46$, $p<0.001$) and a near significant interaction for tobacco use (Wald $X^2(3)=7.76$, $p=0.051$), further analysis on health behaviours was stratified by gender (see Tables 3 and 4).

Differences in tobacco and hazardous alcohol use as a function of sexual orientation identities appeared more pronounced among women than men (Figure 1A and 1B) as indicated by a better fit of the simple model for women than men regarding both tobacco (Akaike Information Criterion (AIC) 35.7 vs 36.4) and hazardous alcohol use (AIC 33.4 vs 35.9).

For peer review only - http://bmjopen.bmj.com/site/about/guidelines.xhtml

For peer review only

Table 2: Sociodemographic characteristics of sample by sexual orientation identity

Characteristic	Total (n=44,030)	Heterosexual (n=41,058)	Bisexual (n=316)	Lesbian/Gay (n=498)	Prefer-not-to-say (n=2,158)	P-value
Mean (SD) Age (years)	47.1 (18.6)	47.3 (18.6)[a]	32.5 (14.6)[b]	39.0 (15.4)[c]	47.6 (19.1)[a]	<0.001
% (N) Women	50.9 (22,406)	51.1 (20,962)[a]	55.7 (176)[a]	37.1 (185)[b]	50.2 (1,083)[a]	<0.001
% (N) White	86.7 (38,192)	87.0 (35,713)[a]	87.4 (277)[a]	96.0 (478)[b]	79.9 (1,724)[c]	<0.001
% (N) Single	41.4 (18,244)	40.4 (16,600)[a]	64.4 (204)[b]	55.6 (277)[b,c]	53.9 (1,163)[c]	<0.001
% (N) Social grade C2DE	45.4 (19.971)	45.1 (18,533)[a]	52.7 (167)[b]	33.4 (166)[c]	51.2 (1,105)[b]	<0.001
% (N) No post-16 qualification	34.3 (15,082)	34.5 (14,156)[a]	28.4 (90)[a]	19.5 (97)[b]	34.2 (739)[a]	<0.001
% (N) With disability	10.3 (4,541)	10.2 (4,188)[a]	19.9 (63)[b]	13.5 (67)[a]	10.3 (223)[a]	<0.001
% (N) Region		[a]	[a]	[b]	[c]	<0.001
North	28.8 (12,665)	29.3 (12,043)	33.4 (106)	32.5 (162)	16.4 (354)	
Central	30.0 (13,209)	29.8 (12,243)	31.5 (100)	18.9 (94)	35.8 (772)	
South	41.2 (18,158)	40.9 (16,773)	35.0 (110)	48.6 (242)	47.8 (1,032)	
% (N) Internet access		[a]	[b]	[c]	[d]	<0.001
Never	12.8 (5,651)	12.7 (5,231)	9.5 (30)	3.4 (17)	17.3 (373)	
≤ Daily	19.8 (8,718)	20.0 (8,218)	12.0 (38)	11.0 (55)	18.9 (407)	
> Daily	67.4 (29,661)	67.2 (27,609)	78.5 (248)	85.5 (426)	63.9 (1,378)	

[a,b,c,d]Different letters for groups in each row indicate significant differences between these groups after controlling for multiple comparisons (p<0.05), same letters indicate no group differences; please note that weighted data are shown

For peer review only

Irrespective of gender, tobacco use was most prevalent among bisexual participants who were nearly twice as likely to smoke as their heterosexual counterparts (for men: 30.7%, 95%CI 23.7%-30.7% vs 20.4%, 95%CI 19.9%-21.0%; PR 1.61, 95%CI 1.28-2.03; for women: 32.4%, 95%CI 25.9%-39.6% vs 17.5%, 95%CI 17.0%-18.0%; PR 1.87, 95%CI 1.52-2.29). Similarly, those who self-identified as lesbian/gay were also more likely to smoke than those with a heterosexual identity (for men: 25.9%, 95%CI 21.3%-31.0%; PR 1.30, 95%CI 1.08-1.56; for women: 24.9%, 95%CI 19.2%-32.6%; PR 1.41, 95%CI 1.11-1.81). However, while for male prefer-not-to-say participants tobacco use prevalence was similar to heterosexual participants (22.7%, 95%CI 20.3%-25.3%; PR 1.07, 95%CI 0.96-1.20), it was significantly lower for female prefer-not-to-say participants (15.5%, 95%CI 13.5%-17.8%; PR 0.87, 95%CI 0.76-1.00; Figure 1A).

The pattern for hazardous alcohol across sexual orientation identities also differed between men and women (Figure 1B). Irrespective of gender, prefer-not-to-say participants had the lowest prevalence of hazardous alcohol use, at nearly half the prevalence observed among heterosexual participants (for men: 13.7%; 95%CI 11.8-16.0% vs 18.4%, 95%CI 17.9%-18.9%; PR 0.74, 95%CI 0.63-0.86; for women: 4.1%, 95%CI 3.0%-5.4% vs 8.3%, 95%CI 7.9%-8.7%; PR 0.48, 95%CI 0.35-0.64). However, while for men hazardous alcohol use was greatest among those who self-identified as gay, at nearly twice the prevalence of heterosexual men (30.0%, 95%CI 25.2%-35.3%; PR 1.61, 95%CI 1.35-1.91), followed by bisexual men (24.3%, 95%CI 17.9%-32.1%; PR 1.41, 95%CI 1.07-1.85), a different pattern was observed in women. Bisexual women had the highest prevalence rates, being more than three times as likely as heterosexual women to engage in hazardous alcohol use (24.4%, 95%CI 18.7%-31.3%; PR 3.08, 95%CI 2.39-3.95), followed by lesbian participants (19.0%, 95%CI 14.0%-25.3%; PR 2.18, 95%CI 1.60-2.96).

Tables 3A and 3B provide information about tobacco use and hazardous alcohol use characteristics by sexual orientation identity in women and men. Among current or recent tobacco users, bisexual women appeared less dependent than heterosexual women (as measured by the heaviness of smoking index), were more likely to smoke roll-your-own cigarettes and consequently spent less money on tobacco than other groups (Table 3A). There were no group differences in hazardous alcohol use characteristics among women.

For peer review only - http://bmjopen.bmj.com/site/about/guidelines.xhtml

For peer review only

Table 3A: Tobacco and hazardous alcohol use characteristics by sexual orientation identity in women

	Total	Heterosexual	Bisexual	Lesbian	Prefer-not-to-say	P-value
Tobacco users*	(n=4,243)	(n=3,952)	(n=60)	(n=50)	(n=181)	
Mean (SD) Cigarettes per day[†]	10.8 (7.7)	10.8 (7.6)	8.1 (7.8)	10.8 (10.3)	10.8 (8.5)	0.091
% (N) Primarily RYO use[†]	43.1 (1,532)	42.4 (1,406)[a]	65.3 (32)[b]	50.0 (20)[a,b]	48.1 (74)[a,b]	0.005
Mean (SD) HSI	1.8 (1.5)	1.8 (1.5)[a]	1.2 (1.4)[b]	1.6 (1.9)[a,b]	1.7 (1.4)[a,b]	0.011
Mean (SD) Urge to smoke	2.9 (1.2)	2.9 (1.2)	2.6 (1.1)	2.8 (1.3)	2.7 (1.1)	0.036
Mean (SD) MTSS[‡]	3.3 (2.0)	3.3 (2.0)	3.3 (2.0)	3.6 (2.0)	3.2 (2.0)	0.775
Mean (SD) Spent per week (£)[‡]	22.5 (19.4)	22.7 (19.4)[a]	14.4 (12.9)[b]	26.5 (27.9)[a]	20.4 (17.0)[a,b]	0.003
% (N) Quit attempt last year	36.0 (1,489)	36.3 (1,397)	31.7 (19)	40.0 (20)	29.9 (53)	0.281
% (N) GP advice	39.9 (1,691)	40.2 (1,587)	25.4 (15)	32.0 (16)	40.3 (73)	0.087
Hazardous alcohol users[§]	(n=4,351)	(n=4,100)	(n=59)	(n=74)	(n=118)	
Mean (SD) Urge to drink	1.5 (0.9)	1.5 (0.9)	1.6 (1.2)	1.8 (1.2)	1.7 (1.0)	0.011
Mean (SD) Motivation to cut down drinking	1.9 (1.6)	1.8 (1.6)	1.9 (1.4)	2.1 (1.9)	2.1 (1.9)	0.184
Mean (SD) Spent per week (£)	14.7 (14.1)	14.6 (14.0)	16.5 (16.6)	19.0 (16.5)	14.8 (11.4)	0.050
% (N) Attempt to cut-down last year	16.3 (676)	16.1 (629)	16.1 (9)	20.3 (15)	21.7 (23)	0.355
% (N) GP advice	7.3 (318)	7.3 (301)	10.2 (6)	6.8 (5)	5.0 (6)	0.647

*Current or past year tobacco users; [†]Current or past year cigarette smokers only; [‡]Current tobacco users only; [§]AUDIT score≥8 or AUDIT-C≥5 score
RYO – roll-your-own; HSI – Heaviness of smoking index; MTSS-Motivation to Stop Scale; [a,b,c,d]Different letters for groups in each row indicate significant differences between these groups after controlling for multiple comparisons ($p<0.05$), same letters indicate no group differences; please note that weighted data are shown

For peer review only - http://bmjopen.bmj.com/site/about/guidelines.xhtml

Table 3B: Tobacco and hazardous alcohol use characteristics by sexual orientation identity in men

	Total	Heterosexual	Bisexual	Gay	Prefer-not-to-say	P-value
Tobacco users*	(n=4,834)	(n=4,426)	(n=51)	(n=87)	(n=269)	
Mean (SD) Cigarettes per day[†]	11.8 (8.7)	11.8 (8.6)	14.3 (17.9)	11.5 (10.1)	11.5 (8.1)	0.256
% (N) Primarily RYO use[†]	51.0 (2,034)	50.7 (1,858)	60.0 (21)	42.7 (32)	58.0 (123)	0.057
Mean (SD) HSI	1.8 (1.5)	1.8 (1.5)	2.1 (1.8)	1.9 (1.7)	1.9 (1.5)	0.670
Mean (SD) Urge to smoke	2.8 (1.2)	2.8 (1.2)	2.6 (1.2)	3.0 (1.1)	2.9 (1.2)	0.103
Mean (SD) MTSS[‡]	3.1 (1.9)	3.1 (1.9)	3.2 (2.2)	3.1 (2.0)	2.9 (1.9)	0.344
Mean (SD) Spent per week (£)[‡]	23.0 (21.4)	23.2 (21.6)	20.3 (18.4)	21.9 (17.7)	21.0 (20.0)	0.350
% (N) Quit attempt last year	32.1 (1,508)	32.1 (1,380)	41.2 (21)	39.5 (34)	28.2 (73)	0.115
% (N) GP advice	33.1 (1,600)	32.8 (1,450)	48.1 (25)	32.2 (28)	36.1 (97)	0.086
Hazardous alcohol users[§]	(n=7,768)	(n=7,285)	(n=58)	(n=154)	(n=272)	
Mean (SD) Urge to drink	1.7 (1.0)	1.7 (1.0)[a]	2.0 (1.3)[a,b]	1.7 (1.2)[a,b]	2.0 (1.0)[b]	<0.001
Mean (SD) Motivation to cut down drinking	1.7 (1.4)	1.7 (1.4)[a]	1.9 (1.7)[a,b]	2.1 (1.7)[b]	1.7 (1.4)[a,b]	0.005
Mean (SD) Spent per week (£)	21.8 (20.2)	21.6 (20.2)	21.3 (21.5)	25.4 (23.0)	24.2 (17.1)	0.027
% (N) Attempt to cut-down last year	14.5 (1,086)	14.5 (1,009)[a]	10.5 (6)[a,b]	23.3 (35)[b]	14.5 (36)[a,b]	0.019
% (N) GP advice	9.9 (766)	10.1 (734)	8.6 (5)	8.4 (13)	5.2 (14)	0.056

*Current or past year tobacco users; [†]Current or past year cigarette smokers only; [‡]Current tobacco users only; [§]AUDIT score≥8 or AUDIT-C≥5 score
RYO – roll-your-own; HSI – Heaviness of smoking index; MTSS-Motivation to Stop Scale; [a,b,c,d]Different letters for groups in each row indicate significant differences between these groups after controlling for multiple comparisons (p<0.05), same letters indicate no group differences; please note that weighted data are shown

For peer review only - http://bmjopen.bmj.com/site/about/guidelines.xhtml

By contrast, among men, there were notable differences between sexual orientation identities in hazardous alcohol use but not tobacco use characteristics (Table 3B). Hazardous drinkers in the prefer-not-to-say group reported greater urges to drink, and gay men reported greater motivation to cut-down on alcohol and had made more attempts to cut-down in the last year compared with heterosexual men.

Are differences in tobacco and hazardous alcohol use prevalence, if any, between the LGB and heterosexual population attenuated when controlling for other covariates?

The associations of sexual orientation identity with tobacco and hazardous alcohol use were re-examined in women and men, controlling for sociodemographic variables (Tables 4A and 4B). Younger age, being white, single, from a lower SES group, lacking post-16 qualifications, reporting a disability and living in Northern England were all independently associated with current tobacco use as was, in men only, lack of internet access. As determined by stepwise forward and backward logistic regression, the association between sexual orientation with smoking status was removed in both women and men after including age, ethnicity, marital status (for women only), SES status and educational attainment into the model; variables are listed in order of impact on changes in variance explained.

By contrast, the association of sexual orientation identity with hazardous alcohol use persisted in women but less so among men, even after controlling for socio-demographic characteristics (Table 4). Compared with heterosexual women, women who self-identified as bisexual (PR 1.63; 95%CI 1.30-2.04) or lesbian (PR 1.37; 95%CI 1.02-1.82) were more likely to engage in hazardous alcohol use in adjusted analysis, while prefer-not-to-say women were less likely to do so (PR 0.61; 95%CI 0.45-0.81; Table 4A). By contrast, among men, differences only persisted for prefer-not-to-say men who remained less likely to engage in hazardous drinking (PR 0.82; 95%CI 0.71-0.95) than heterosexuals. However, bisexual (PR 1.07, 95%CI 0.83-1.38) and gay men (OR 1.10, 95%CI 0.94-1.30) no longer differed from heterosexual men in hazardous drinking after adjusting for sociodemographic covariates (Table 4B). Being younger, white, single, from a higher SES group and having post-16 qualifications as well as accessing the internet and

For peer review only - http://bmjopen.bmj.com/site/about/guidelines.xhtml

being from Northern England were all independently associated with hazardous drinking in both men and women.

For peer review only

For peer review only - http://bmjopen.bmj.com/site/about/guidelines.xhtml

Table 4A: Factors associated with tobacco and hazardous alcohol use in women

Factor	Tobacco use				Hazardous alcohol use			
	PR (95%CI)	P-value	aPR (95%CI)	P-value	PR (95%CI)	P-value	aPR (95%CI)	P-value
Age	0.99 (0.98-0.99)	<0.001	0.98 (0.98-0.98)	<0.001	0.97 (0.97-0.97)	<0.001	0.98 (0.97-0.98)	<0.001
White	2.97 (2.59-3.42)	<0.001	3.57 (3.10-4.11)	<0.001	4.14 (3.24-5.29)	<0.001	4.90 (3.82-6.28)	<0.001
Single	1.51 (1.43-1.59)	<0.001	1.27 (1.20-1.34)	<0.001	1.49 (1.37-1.63)	<0.001	1.47 (1.35-1.59)	<0.001
Social grade C2DE	2.01 (1.94-2.17)	<0.001	1.69 (1.59-1.80)	<0.001	0.81 (0.74-0.88)	<0.001	0.85 (0.78-0.93)	<0.001
No post-16 qualification	1.57 (1.49-1.66)	<0.001	1.39 (1.31-1.47)	<0.001	0.59 (0.54-0.65)	<0.001	0.80 (0.73-0.89)	<0.001
With disability	1.39 (1.29-1.50)	<0.001	1.33 (1.24-1.43)	<0.001	0.84 (0.73-0.97)	0.019	1.13 (0.98-1.30)	0.092
Region								
North (Ref)	1	<0.001	1	<0.001	1	<0.001	1	<0.001
Central	0.74 (0.69-0.79)		0.83 (0.78-0.89)		0.37 (0.33-0.41)		0.45 (0.40-0.51)	
South	0.67 (0.63-0.71)		0.86 (0.81-0.91)		0.47 (0.42-0.51)		0.59 (0.53-0.64)	
Internet access								
Never (Ref)	1	0.125	1	0.063	1	<0.001	1	<0.001
≤ Daily	0.95 (0.86-1.04)		0.94 (0.86-1.04)		2.08 (1.62-2.66)		1.47 (1.14-1.89)	
> Daily	1.02 (0.94-1.10)		0.89 (0.81-0.99)		4.73 (3.80-5.89)		2.26 (1.78-2.88)	
Sexual orientation identity								
Heterosexual (Ref)	1		1		1		1	
Bisexual	1.87 (1.52-2.29)	<0.001	1.04 (0.88-1.24)	0.466	3.08 (2.39-3.95)	<0.001	1.63 (1.30-2.04)	<0.001
Lesbian	1.41 (1.11-1.81)		1.14 (0.93-1.39)		2.18 (1.60-2.96)		1.37 (1.02-1.82)	
Prefer-not-to-say	0.87 (0.76-1.00)		0.94 (0.83-1.08)		0.48 (0.35-0.64)		0.61 (0.45-0.81)	

PR-prevalence (risk) ratio, aPR – adjusted prevalence (risk) ratio

For peer review only - http://bmjopen.bmj.com/site/about/guidelines.xhtml

Table 4B: Factors associated with tobacco and hazardous alcohol use in men

Factor	Tobacco use				Hazardous alcohol use			
	PR (95%CI)	P-value	aPR (95%CI)	P-value	PR (95%CI)	P-value	aPR (95%CI)	P-value
Age	0.98 (0.98-0.99)	<0.001	0.98 (0.98-0.98)	<0.001	0.99 (0.98-0.99)	<0.001	0.99 (0.98-0.99)	<0.001
White	1.19 (1.10-1.28)	<0.001	1.44 (1.34-1.55)	<0.001	5.53 (4.68-6.54)	<0.001	6.04 (5.10-7.15)	<0.001
Single	1.62 (1.54-1.70)	<0.001	1.16 (1.10-1.23)	<0.001	1.37 (1.30-1.45)	<0.001	1.21 (1.14-1.28)	<0.001
Social grade C2DE	1.93 (1.83-2.04)	<0.001	1.55 (1.47-1.65)	<0.001	0.81 (0.77-0.86)	<0.001	0.87 (0.82-0.92)	<0.001
No post-16 qualification	1.62 (1.54-1.70)	<0.001	1.38 (1.31-1.46)	<0.001	0.77 (0.72-0.82)	<0.001	0.88 (0.82-0.94)	<0.001
With disability	1.39 (1.29-1.50)	<0.001	1.35 (1.26-1.45)	<0.001	0.91 (0.82-1.00)	0.059	1.01 (0.92-1.12)	0.797
Region								
North (Ref)	1		1		1		1	
Central	0.85 (0.76-0.91)	<0.001	0.89 (0.84-0.95)	0.001	0.46 (0.43-0.50)	<0.001	0.54 (0.50-0.58)	<0.001
South	0.81 (0.75-0.87)		0.94 (0.89-1.00)		0.61 (0.57-0.65)		0.70 (0.66-0.75)	
Internet access								
Never (Ref)	1		1		1		1	
≤ Daily	0.83 (0.77-0.91)	<0.001	0.83 (0.76-0.90)	<0.001	1.34 (1.17-1.54)	<0.001	1.09 (0.95-1.25)	<0.001
> Daily	0.86 (0.80-0.92)		0.76 (0.70-0.82)		1.94 (1.73-2.19)		1.31 (1.15-1.50)	
Sexual orientation identity								
Heterosexual (Ref)	1		1		1		1	
Bisexual	1.61 (1.28-2.03)	<0.001	1.15 (0.93-1.42)	0.517	1.41 (1.07-1.85)	<0.001	1.07 (0.83-1.38)	0.031
Gay	1.30 (1.08-1.56)		1.06 (0.89-1.26)		1.61 (1.35-1.91)		1.10 (0.94-1.30)	
Prefer-not-to-say	1.07 (0.96-1.20)		1.03 (0.92-1.14)		0.74 (0.63-0.86)		0.82 (0.71-0.95)	

PR-prevalence (risk) ratio, aPR – adjusted prevalence (risk) ratio

For peer review only

For peer review only - http://bmjopen.bmj.com/site/about/guidelines.xhtml

Discussion

Our study found that in England, lesbian, gay and bisexual men and women appear to have higher rates of tobacco and hazardous alcohol use. This is in agreement with previous studies of young people and adults, reporting greater risk of sexual minorities engaging in tobacco and hazardous alcohol use than their heterosexual counterparts.[9, 32-34] Those who elected not to disclose their sexual orientation were less likely to engage in these risky health behaviours. In general, it appears that differences were more pronounced among women than men, as has been previously reported.[35] However, contrary to previous work, we found that differences between sexual orientation identities in tobacco use disappeared when socio-demographic variables were taken into account. Disparities in hazardous alcohol use across sexual orientation identities were also somewhat attenuated when controlling for these covariates, but mainly among men, and remained significant in women.

Differences in tobacco use between LGB and heterosexual participants in this study appear to be explained in the most part by underlying variations in major socio-demographic characteristics. However, it is important to remember that tobacco use disparities remain in this group,[10, 32, 36] even if these may not be attributable to factors specifically associated with sexual orientation, and further work needs to be done to support minority groups to reduce tobacco consumption. By contrast, the association of hazardous alcohol use with being a sexual minority largely persisted after controlling for major socio-demographic confounders, but only among women. This persistence suggests that there may exist specific influences that put lesbian and bisexual women at greater risk as has been previously reported for both alcohol use [11, 37] and other health behaviours.[38] While our findings replicate the existing evidence of a higher risk profile in sexual minority groups,[14, 34, 39] they underline the need for gender-specific tailoring of health messages to account for the differences within the LGB community. This is for instance shown by the finding that gay but not lesbian hazardous drinkers reported both greater motivation to cut-down on alcohol and had more quit attempts compared with heterosexual hazardous drinkers.

For peer review only - http://bmjopen.bmj.com/site/about/guidelines.xhtml

The finding that the association of sexual orientation identity with tobacco use was attenuated after controlling for socio-demographic characteristics, in particular age, ethnicity, marital status (among women), socio-economic group and educational attainment, could be due to several reasons. Self-identification as LGB is greater in younger than older age groups [24] as are smoking rates. Participants who identified as lesbian/gay were more likely to be white and LGB participants were more likely to be single, both factors associated with greater smoking rates in the general population.[24, 40] However, it is more difficult to explain the role of deprivation (as measured by education or socio-economic group). Generally, deprivation is strongly and positively associated with tobacco use [41] and the association of greater deprivation with tobacco use was as expected for bisexual participants, particularly bisexual women, who were more likely to smoke, use cheaper roll-your-own cigarettes and spend less on tobacco. Moreover, this group was also younger than other groups, including lesbian/gay participants, which would explain both the association with lower dependence (older smokers are more dependent) and deprivation (age inversely relates to income). By contrast, lesbian/gay participants had both lower levels of deprivation and higher smoking rates than heterosexuals, suggesting that, unlike for other groupings (e.g. by ethnicity or gender [42]), the association of deprivation with tobacco use may be more complicated in sexual orientation minorities. Smoking as a health behaviour may be performed differently in these groups, both compared to each other and in comparison with heterosexuals.[8] This issue deserves further investigation.

More generally, the results may suggest a genuine shift in the environment encountered by sexual minorities. The social-ecological model [43] posits that interactions with the environment determines risk behaviour. Therefore reduced differences in tobacco use could indicate that the environment for sexual minorities and the majority is becoming increasingly similar. Moreover, the introduction of 'smokefree' laws may have had a disproportionately greater impact on tobacco use among sexual minorities as they are more likely to spend time in social, recreational spaces [18] in which smoking used to be the norm.

The persistent association with hazardous alcohol use among sexual minority participants in this study, specifically lesbian and bisexual women, highlights the negative behavioural consequences

For peer review only - http://bmjopen.bmj.com/site/about/guidelines.xhtml

experienced by social minorities, not easily explained by general socio-demographic covariates. One potential explanatory factor not explored in the current analysis is the known increased risk of mental health problems in the LGB community [5] which is also associated with problem drinking.[44] Other factors include the use of recreational spaces by sexual minorities where alcohol drinking may be the norm. However, the finding that the association with hazardous alcohol use is particularly strong among sexual minority women suggests that other gender-specific influences may be at work. For instance, it may be an expression of gender non-conformity (i.e. to go against stereotypical views of female vs. male drinking behaviour [45]) or it may reflect the fact that women are more likely to experience double discrimination [46] which may increase the propensity to engage in risky health behaviours as a coping mechanism.

It is interesting to note that participants who preferred not to disclose their sexual orientation were less likely to engage in hazardous alcohol use, whereas no differences were observed in this group in relation to tobacco use. As there was a larger proportion of non-white participants in this group, this may be explained in part by different cultural and religious norms and stigma imposed on people, i.e. many religions that adopt a negative stance towards sexual minorities, which may lead to non-disclosure of sexual minority identity,[47] often also have punitive views on alcohol, resulting in lower consumption.[48] Similarly, while there are few ethnic and cultural disparities in male (though not female) smoking rates, alcohol use is differently patterned by ethnicity with some ethnic minorities drinking less.[49] Against these barriers to drinking, self-selection may therefore explain why those prefer-not-to-say participants who do drink are more dependent.

This study had a number of limitations. The cross-sectional nature of the design makes it difficult to assess causal pathways. We did attempt to reduce the risk of unmeasured bias by controlling for a range of known socio-demographic confounders for tobacco and hazardous alcohol use. While this sample was representative of the general population of England and results therefore likely generalise to other high-income countries, the subgroup of participants with a minority sexual orientation identity was relatively small and so the study would not have been powered to detect smaller, more subtle differences. As has been

For peer review only - http://bmjopen.bmj.com/site/about/guidelines.xhtml

For peer review only

previously discussed, sexuality can be measured in different ways. Here, a measure of sexual orientation identity was used, as this is argued to be the most relevant dimension to investigate the relationship of sexual orientation with disadvantage.[50] Future research should consider investigating this question using measures of behaviour and attraction: groups who do not choose to identify as LGB but carry out same-sex behaviour for instance, may be quite different. Not everyone is comfortable adopting an identity label and some may have not wanted to select one of the options offered by the ONS question. Lastly, no objective measure of tobacco and alcohol use was taken; however, both behaviours were assessed using validated and reliable scales and low demand, anonymous studies tend to provide relatively unbiased results.[51]

In conclusion, sexual orientation disparities in tobacco and hazardous alcohol use exist in England, with LGB people exhibiting greater levels of risky health behaviours. However, differences in tobacco and hazardous alcohol use appear mainly associated with general socio-demographic differences in men whereas differences in hazardous alcohol use, but not tobacco use, persist in women after controlling for socio-demographic characteristics. Further research is now needed to consider the explanatory factors and to develop interventions to remove health inequalities in these populations.

For peer review only

References

1. Dilley JA, Simmons KW, Boysun MJ, et al. Demonstrating the importance and feasibility of including sexual orientation in public health surveys: health disparities in the Pacific Northwest. *Am J Public Health* 2010;100(3):460-7. doi: 10.2105/AJPH.2007.130336

2. McCabe SE, Hughes TL, Bostwick W, et al. Measurement of sexual identity in surveys: implications for substance abuse research. *Arch Sex Behav* 2012;41(3):649-57. doi: 10.1007/s10508-011-9768-7

3. Haseldon L, Joloza T. Measuring Sexual Identity: A Guide for Researchers. Newport, Wales: Office for National Statistics 2009.

4. Laumann EO, Gagnon JH, Michael RT, et al. The social organization of sexuality: Sexual practices in the United States. Chicago: University of Chicago Press 1994.

5. Semlyen J, King M, Varney J, et al. Sexual orientation and symptoms of common mental disorder or low wellbeing: combined meta-analysis of 12 UK population health surveys. *BMC Psychiatry* 2016;16(1):67. doi: 10.1186/s12888-016-0767-z

6. Cochran SD. Emerging issues in research on lesbians' and gay men's mental health: does sexual orientation really matter? *Am Psychol* 2001;56(11):931-47.

7. Dunne MP, Martin NG, Bailey JM, et al. Participation bias in a sexuality survey: psychological and behavioural characteristics of responders and non-responders. *Int J Epidemiol* 1997;26(4):844-54.

8. Boehmer U, Miao X, Linkletter C, et al. Adult health behaviors over the life course by sexual orientation. *Am J Public Health* 2012;102(2):292-300. doi: 10.2105/AJPH.2011.300334

9. Rosario M, Corliss HL, Everett BG, et al. Sexual orientation disparities in cancer-related risk behaviors of tobacco, alcohol, sexual behaviors, and diet and physical activity: pooled Youth Risk Behavior Surveys. *Am J Public Health* 2014;104(2):245-54. doi: 10.2105/AJPH.2013.301506

10. Blosnich J, Lee JG, Horn K. A systematic review of the aetiology of tobacco disparities for sexual minorities. *Tob Control* 2013;22(2):66-73. doi: 10.1136/tobaccocontrol-2011-050181

11. Bloomfield K, Wicki M, Wilsnack S, et al. International differences in alcohol use according to sexual orientation. *Subst Abus* 2011;32(4):210-9. doi: 10.1080/08897077.2011.598404

12. Chakraborty A, McManus S, Brugha TS, et al. Mental health of the non-heterosexual population of England. *Br J Psychiatry* 2011;198(2):143-8. doi: 10.1192/bjp.bp.110.082271

13. Collier KL, van Beusekom G, Bos HM, et al. Sexual orientation and gender identity/expression related peer victimization in adolescence: a systematic review of associated psychosocial and health outcomes. *J Sex Res* 2013;50(3-4):299-317. doi: 10.1080/00224499.2012.750639

14. King M, Semlyen J, Tai SS, et al. A systematic review of mental disorder, suicide, and deliberate self harm in lesbian, gay and bisexual people. *BMC Psychiatry* 2008;8(1):70. doi: citeulike-article-id:8338079
doi: 10.1186/1471-244x-8-70

15. Hawkins JD, Catalano RF, Miller JY. Risk and protective factors for alcohol and other drug problems in adolescence and early adulthood: implications for substance abuse prevention. *Psychol Bull* 1992;112(1):64-105.

For peer review only - http://bmjopen.bmj.com/site/about/guidelines.xhtml

16. Swendsen J, Conway KP, Degenhardt L, et al. Mental disorders as risk factors for substance use, abuse and dependence: results from the 10-year follow-up of the National Comorbidity Survey. *Addiction* 2010;105(6):1117-28. doi: 10.1111/j.1360-0443.2010.02902.x

17. Abdulrahim D, Whiteley C, Moncrieff M, et al. Club Drug Use Among Lesbian, Gay, Bisexual and Trans (LGBT) People. London: Novel Psychoactive Treatment UK Network (NEPTUNE) 2016.

18. Talley AE, Sher KJ, Littlefield AK. Sexual orientation and substance use trajectories in emerging adulthood. *Addiction* 2010;105(7):1235-45. doi: 10.1111/j.1360-0443.2010.02953.x

19. Talley AE, Sher KJ, Steinley D, et al. Patterns of alcohol use and consequences among empirically derived sexual minority subgroups. *J Stud Alcohol Drugs* 2012;73(2):290-302.

20. Aronsson H, Lind J, Ghirlanda S, et al. Parental influences on sexual preferences: the case of attraction to smoking. *J Evol Psychol* 2011;9 doi: http://dx.doi.org/10.1556/JEP.9.2011.12.1

21. Smith EA, Malone RE. The outing of Philip Morris: advertising tobacco to gay men. *Am J Public Health* 2003;93(6):988-93.

22. Fidler JA, Shahab L, West O, et al. 'The Smoking Toolkit Study': A national study of smoking and smoking cessation in England. *BMC Public Health* 2011;11(1):479.

23. Beard E, Brown J, West R, et al. Protocol for a national monthly survey of alcohol use in England with 6-month follow-up: 'the Alcohol Toolkit Study'. *BMC Public Health* 2015;15:230. doi: 10.1186/s12889-015-1542-7

24. Office for National Statistics. Integrated Household Survey, January to December 2014: Experimental Statistics http://web.ons.gov.uk/ons/dcp171778_418136.pdf (accessed 10/06/2016): ONS; 2015 [

25. Heatherton TF, Kozlowski LT, Frecker RC, et al. Measuring the heaviness of smoking: using self-reported time to the first cigarette of the day and number of cigarettes smoked per day. *Br J Addict* 1989;84(7):791-99.

26. Fidler JA, Shahab L, West R. Strength of urges to smoke as a measure of severity of cigarette dependence: comparison with the Fagerstrom Test for Nicotine Dependence and its components. *Addiction* 2011;106(3):631-38.

27. Kotz D, Brown J, West R. Predictive validity of the Motivation To Stop Scale (MTSS): A single-item measure of motivation to stop smoking. *Drug Alcohol Depend* 2013;128(1-2):15-19.

28. Babor T, HIggins-Biddle J, Saunders J, et al. AUDIT: the Alcohol Use Disorders Identification Test: guidelines for use in primary care. 2nd ed. Geneva, Switzerland: WHO 2001.

29. National Readership Survey. http://www.nrs.co.uk/nrs-print/methodology/technical-information/ (accessed 23.01.15). 2015

30. Brown J, West R, Angus C, et al. Comparison of brief interventions in primary care on smoking and excessive alcohol consumption: a population survey in England. *Br J Gen Pract* 2016;66(642):e1-9. doi: 10.3399/bjgp16X683149

31. Benjamini Y, Hochberg Y. Controlling the False Discovery Rate - A Practical and Powerful Approach to Multiple Testing. *J R Statist Soc, B* 1995;57(1):289-300.

32. Lee JG, Griffin GK, Melvin CL. Tobacco use among sexual minorities in the USA, 1987 to May 2007: a systematic review. *Tob Control* 2009;18(4):275-82. doi: 10.1136/tc.2008.028241

For peer review only - http://bmjopen.bmj.com/site/about/guidelines.xhtml

33. Ryan H, Wortley PM, Easton A, et al. Smoking among lesbians, gays, and bisexuals: a review of the literature. *Am J Prev Med* 2001;21(2):142-9.

34. Hagger-Johnson G, Taibjee R, Semlyen J, et al. Sexual orientation identity in relation to smoking history and alcohol use at age 18/19: cross-sectional associations from the Longitudinal Study of Young People in England (LSYPE). *BMJ Open* 2013;3(8):e002810. doi: 10.1136/bmjopen-2013-002810

35. McCabe SE, Hughes TL, Bostwick WB, et al. Sexual orientation, substance use behaviors and substance dependence in the United States. *Addiction* 2009;104(8):1333-45. doi: 10.1111/j.1360-0443.2009.02596.x

36. Johnson SE, Holder-Hayes E, Tessman GK, et al. Tobacco Product Use Among Sexual Minority Adults: Findings From the 2012-2013 National Adult Tobacco Survey. *Am J Prev Med* 2016;50(4):e91-e100. doi: 10.1016/j.amepre.2015.07.041

37. Midanik L, Drabble L, Trocki K, et al. Sexual orientation and alcohol use: identiy versus behavior measures. *Journal of LGBT Research* 2006;3:25-35. doi: 10.1300/J463v03n01_04

38. Fallin A, Goodin A, Lee YO, et al. Smoking characteristics among lesbian, gay, and bisexual adults. *Prev Med* 2015;74:123-30. doi: 10.1016/j.ypmed.2014.11.026

39. Branstrom R, Hatzenbuehler ML, Pachankis JE. Sexual orientation disparities in physical health: age and gender effects in a population-based study. *Soc Psychiatry Psychiatr Epidemiol* 2016;51(2):289-301. doi: 10.1007/s00127-015-1116-0

40. Office for National Statistics. Adult Smoking Habits in Great Britain, 2013 http://www.ons.gov.uk/peoplepopulationandcommunity/healthandsocialcare/healthandlifeexpectancies/compendium/opinionsandlifestylesurvey/2015-03-19/adultsmokinghabitsingreatbritain2013/pdf (accessed 10/06/2016)2015 [

41. Jarvis MJ, Wardle J. Social patterning of individual health behaviours: the case of cigarette smoking. In: Marmot M, Wilkinson RG, eds. Social Determinants of Health. New York: Oxford University Press 2005:224-37.

42. Barbeau EM, Krieger N, Soobader MJ. Working class matters: socioeconomic disadvantage, race/ethnicity, gender, and smoking in NHIS 2000. *AmJ Public Health* 2004;94(2):269-78.

43. Bronfenbrenner U. The ecology of human development: Experiments by nature and design. Cambridge, MA: Havard University Press 1979.

44. Makela P, Raitasalo K, Wahlbeck K. Mental health and alcohol use: a cross-sectional study of the Finnish general population. *Eur J Public Health* 2015;25(2):225-31. doi: 10.1093/eurpub/cku133

45. Rosario M, Schrimshaw EW, Hunter J. Butch/Femme differences in substance use and abuse among young lesbian and bisexual women: examination and potential explanations. *Subst Use Misuse* 2008;43(8-9):1002-15. doi: 10.1080/10826080801914402

46. Bowleg L, Huang J, Brooks K, et al. Triple jeopardy and beyond: mulitple minority stress and resilience among black lesbians. *Journal of Lesbian Studies* 2003;7:87-108.

47. Schrimshaw EW, Downing MJ, Jr., Cohn DJ. Reasons for Non-Disclosure of Sexual Orientation Among Behaviorally Bisexual Men: Non-Disclosure as Stigma Management. *Arch Sex Behav* 2016 doi: 10.1007/s10508-016-0762-y

For peer review only - http://bmjopen.bmj.com/site/about/guidelines.xhtml

48. Michalak L, Trocki K, Bond J. Religion and alcohol in the U.S. National Alcohol Survey: how important is religion for abstention and drinking? *Drug Alcohol Depend* 2007;87(2-3):268-80. doi: 10.1016/j.drugalcdep.2006.07.013

49. Beard E, Brown J, West R, et al. Healthier central England or North-South divide? Analysis of national survey data on smoking and high-risk drinking. *BMJ Open* 2017;7(3):e014210. doi: 10.1136/bmjopen-2016-014210

50. Vizard T. Measuring sexual identity in the United Kingdom. *Journal of Bisexuality* 2014;14:524-43. doi: 10.1080/15299716.2914.9311830

51. Bonevski B, Campbell E, Sanson-Fisher RW. The validity and reliability of an interactive computer tobacco and alcohol use survey in general practice. *Addict Behav* 2010;35(5):492-8. doi: 10.1016/j.addbeh.2009.12.030

For peer review only

For peer review only - http://bmjopen.bmj.com/site/about/guidelines.xhtml

Acknowledgement

None.

Author contributions statement

CM, JB & RW conceived the original idea for this study. CM obtained funding. JB and RW managed the day-to-day running of the study. LS undertook the data analyses and wrote the initial draft with further input from all authors. LS is guarantor for this article. LS, JB, GHJ, SM, JS, RW and CM read, reviewed and approved the final version. All researchers listed as authors are independent from the funders and all final decisions about the research were taken without constraint by the investigators. LS had full access to all the data in the study and had final responsibility for the decision to submit for publication.

Additional Information

Funding

We are grateful to Cancer Research UK who funded this analysis (C52095/A18725). The Smoking Toolkit Study is primarily funded by Cancer Research UK (C1417/A14135; C36048/A11654; C44576/A19501; C1417/A22962) and has previously also been funded by Pfizer, GSK, and the Department of Health. The National Institute for Health Research (NIHR) School for Public Health Research funded data collection for the Alcohol Toolkit Study (SPHR-SWP-ALC-WP5). JB and RW are funded by Cancer Research UK (C1417/A22962). SPHR is a partnership between the Universities of Sheffield; Bristol; Cambridge; Exeter; UCL; The London School for Hygiene and Tropical Medicine; the LiLaC collaboration between the Universities of Liverpool and Lancaster and Fuse; The Centre for Translational Research in Public Health, a collaboration between Newcastle, Durham, Northumbria, Sunderland and Teesside Universities. The views expressed are those of the authors(s) and not necessarily those of the NHS, NIHR, or Department of Health.

Conflict of Interest

For peer review only

LS has received a research grant and honoraria for a talk and travel expenses from a Pfizer, manufacturer of smoking cessation medications. JB has received unrestricted research funding from Pfizer to study smoking cessation. RW has received travel funds and hospitality from, and undertaken research and consultancy for, pharmaceutical companies that manufacture or research products aimed at helping smokers to stop. GHJ receives royalties from ''Introduction to Research Methods and Data Analysis in Psychology'' (2013) and 'Psychometric Assessment, Statistics and Report Writing' (2013), both published by Pearson and 'Introduction to Research Methods and Data Analysis in the Health Sciences'. He has also worked as a consultant for various universities and Public Health England. His substantive contribution to the manuscript was prior to June 2016. CM, SM and JS have no competing interests.

Data sharing

Headline data from the Toolkit Study are published monthly at http://www.smokinginengland.info/. Data will be made available upon request.

For peer review only

For peer review only - http://bmjopen.bmj.com/site/about/guidelines.xhtml

Figure legend

Figure 1: PNTS – Prefer-not-to-say; Error bars are 95% confidence intervals; All data are unadjusted

For peer review only

For peer review only - http://bmjopen.bmj.com/site/about/guidelines.xhtml

Figure 1: Prevalence of (A) tobacco use and (B) hazardous alcohol use by gender and sexual orientation identity

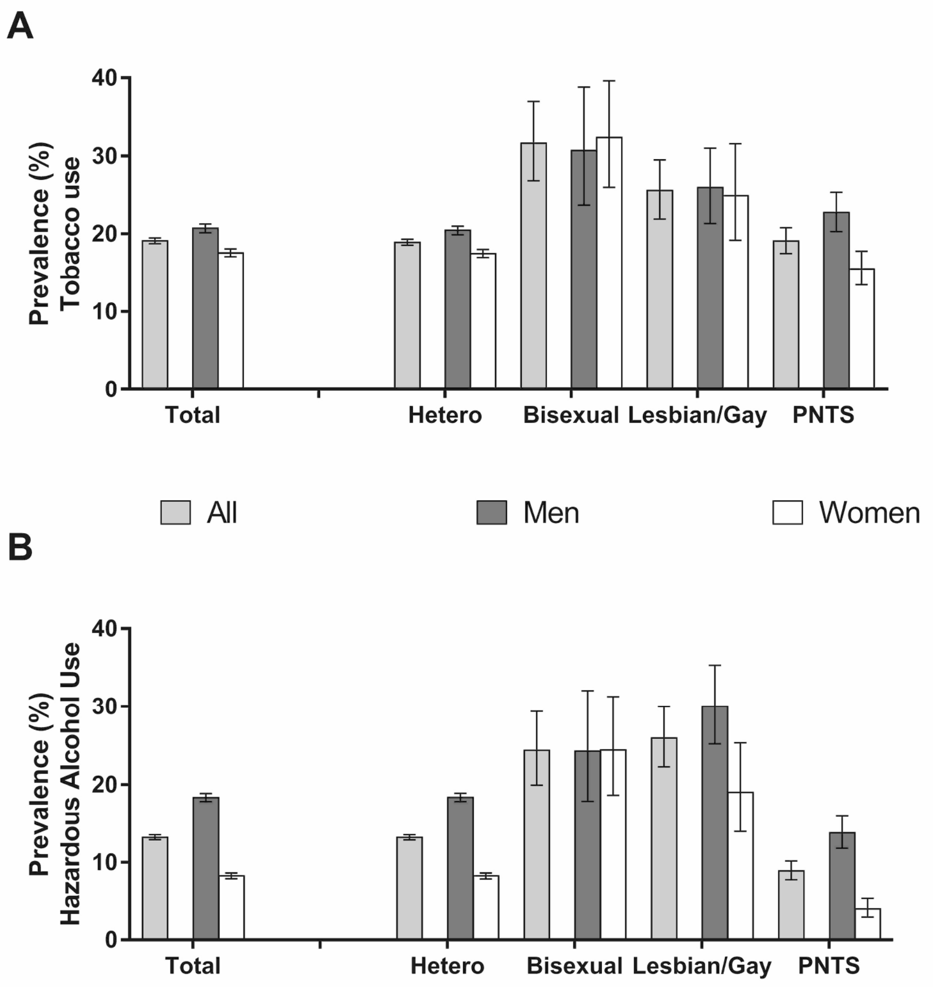

218x257mm (300 x 300 DPI)

STROBE Statement—checklist of items that should be included in reports of observational studies

	Item No	Recommendation
**Title and abstract**	1	(*a*) Indicate the study's design with a commonly used term in the title or the abstract **Page 1**
		(*b*) Provide in the abstract an informative and balanced summary of what was done and what was found **Page 2**
**Introduction**		
Background/rationale	2	Explain the scientific background and rationale for the investigation being reported **Page 4-5**
Objectives	3	State specific objectives, including any prespecified hypotheses **Page 6**
**Methods**		
Study design	4	Present key elements of study design early in the paper **Page 7**
Setting	5	Describe the setting, locations, and relevant dates, including periods of recruitment, exposure, follow-up, and data collection **Page 7**
Participants	6	(*a*) *Cohort study*—Give the eligibility criteria, and the sources and methods of selection of participants. Describe methods of follow-up<br>*Case-control study*—Give the eligibility criteria, and the sources and methods of case ascertainment and control selection. Give the rationale for the choice of cases and controls<br>*Cross-sectional study*—Give the eligibility criteria, and the sources and methods of selection of participants **Page 7**
		(*b*) *Cohort study*—For matched studies, give matching criteria and number of exposed and unexposed<br>*Case-control study*—For matched studies, give matching criteria and the number of controls per case
Variables	7	Clearly define all outcomes, exposures, predictors, potential confounders, and effect modifiers. Give diagnostic criteria, if applicable **Page 7-9**
Data sources/ measurement	8*	For each variable of interest, give sources of data and details of methods of assessment (measurement). Describe comparability of assessment methods if there is more than one group **Page 7-9**
Bias	9	Describe any efforts to address potential sources of bias **Page 9**
Study size	10	Explain how the study size was arrived at **N/A**
Quantitative variables	11	Explain how quantitative variables were handled in the analyses. If applicable, describe which groupings were chosen and why **Page 9**
Statistical methods	12	(*a*) Describe all statistical methods, including those used to control for confounding **Page 9**
		(*b*) Describe any methods used to examine subgroups and interactions **Page 9**
		(*c*) Explain how missing data were addressed **Page 9**
		(*d*) *Cohort study*—If applicable, explain how loss to follow-up was addressed<br>*Case-control study*—If applicable, explain how matching of cases and controls was addressed<br>*Cross-sectional study*—If applicable, describe analytical methods taking account of sampling strategy **Page 9**
		(*e*) Describe any sensitivity analyses **N/A**

Continued on next page

For peer review only

**Results**		
Participants	13*	(a) Report numbers of individuals at each stage of study—eg numbers potentially eligible, examined for eligibility, confirmed eligible, included in the study, completing follow-up, and analysed **Page 7/10 and Table 2**
		(b) Give reasons for non-participation at each stage **N/A**
		(c) Consider use of a flow diagram **N/A**
Descriptive data	14*	(a) Give characteristics of study participants (eg demographic, clinical, social) and information on exposures and potential confounders **Page 10 / Tables 1 and 2 / Figure 1**
		(b) Indicate number of participants with missing data for each variable of interest **N/A**
		(c) *Cohort study*—Summarise follow-up time (eg, average and total amount)
Outcome data	15*	*Cohort study*—Report numbers of outcome events or summary measures over time
		Case-control study—Report numbers in each exposure category, or summary measures of exposure
		Cross-sectional study—Report numbers of outcome events or summary measures **Page 10 / Table 1 and Figure 1**
Main results	16	(*a*) Give unadjusted estimates and, if applicable, confounder-adjusted estimates and their precision (eg, 95% confidence interval). Make clear which confounders were adjusted for and why they were included **Page 10,12, 15 Table 1 and Figure 1**
		(*b*) Report category boundaries when continuous variables were categorized **N/A**
		(*c*) If relevant, consider translating estimates of relative risk into absolute risk for a meaningful time period **N/A**
Other analyses	17	Report other analyses done—eg analyses of subgroups and interactions, and sensitivity analyses **Page 10-17**
**Discussion**		
Key results	18	Summarise key results with reference to study objectives **Page 18**
Limitations	19	Discuss limitations of the study, taking into account sources of potential bias or imprecision. Discuss both direction and magnitude of any potential bias **Page 20/21**
Interpretation	20	Give a cautious overall interpretation of results considering objectives, limitations, multiplicity of analyses, results from similar studies, and other relevant evidence **Page 18-21**
Generalisability	21	Discuss the generalisability (external validity) of the study results **Page 20**
**Other information**		
Funding	22	Give the source of funding and the role of the funders for the present study and, if applicable, for the original study on which the present article is based **Page 26**

*Give information separately for cases and controls in case-control studies and, if applicable, for exposed and unexposed groups in cohort and cross-sectional studies.

Note: An Explanation and Elaboration article discusses each checklist item and gives methodological background and published examples of transparent reporting. The STROBE checklist is best used in conjunction with this article (freely available on the Web sites of PLoS Medicine at http://www.plosmedicine.org/, Annals of Internal Medicine at http://www.annals.org/, and Epidemiology at http://www.epidem.com/). Information on the STROBE Initiative is available at www.strobe-statement.org.

For peer review only

For peer review only - http://bmjopen.bmj.com/site/about/guidelines.xhtml
